# Concerns for efficacy of a 30-valent M-protein-based *Streptococcus pyogenes* vaccine in regions with high rates of rheumatic heart disease

**DOI:** 10.1371/journal.pntd.0007511

**Published:** 2019-07-03

**Authors:** Philip M. Giffard, Steven Y. C. Tong, Deborah C. Holt, Anna P. Ralph, Bart J. Currie

**Affiliations:** 1 Menzies School of Health Research, Division of Global and Tropical Health, Darwin, Australia; 2 College of Health and Human Sciences, Charles Darwin University, Darwin, Australia; 3 Victorian Infectious Disease Service, The Royal Melbourne Hospital, and Doherty Department University of Melbourne, at the Peter Doherty Institute for Infection and Immunity, Melbourne, Victoria, Australia; 4 Division of Medicine, Royal Darwin Hospital, Darwin, Australia; Yale School of Public Health, UNITED STATES

## Abstract

The prevalence of rheumatic heart disease (RHD) in the Aboriginal population of the Australian Northern Territory is high, and *Streptococcus pyogenes* skin infections likely contribute to this. A promising candidate *S*. *pyogenes* “30mer” vaccine is composed of 30 pharyngitis associated type-specific antigens from the *S*. *pyogenes* M protein. Cross opsonisation experiments suggest that 30mer vaccine protection may extend to non-cognate *emm* types. A new “*emm* cluster” scheme for classifying M protein is based on the full-length coding sequence, and correlates with functional and immunological properties, and anatomical tropism. Twenty-seven years of research in the Northern Territory has yielded 1810 *S*. *pyogenes* isolates with clinical and *emm* type data. The primary aim was to analyse these data with reference to the *emm* cluster scheme and cross opsonisation information, to inform estimation of 30mer vaccine efficacy in the Northern Territory. The isolates encompass 101 *emm* types. Variants of cluster A-C were enriched in throat isolates, and variants of *emm* cluster D enriched in skin isolates. Throat isolates were enriched for 30mer vaccine cognate *emm* types in comparison with skin isolates of which only 25% were vaccine *emm* types. While cross opsonisation data indicates potential for enhancing 30mer vaccine coverage, more than one third of skin isolates were within 38 *emm* types untested for cross opsonisation. *Emm* cluster D variants, in particular *emm* cluster D4, were not only all non-cognate with the vaccine, but were abundant and diverse, and less likely to be cross-opsonisation positive than other *emm* clusters. Long term persistence of many *emm* types in the study area was revealed. It was concluded that the 30mer vaccine efficacy in the Northern Territory will likely require both cross protection, and additional measures to elicit immunity against variants of *emm* cluster D.

## Author summary

The bacterium *Streptococcus pyogenes* causes throat and skin infections. A danger from such infections is an immune response that attacks human heart tissue, leading to rheumatic heart disease, which is difficult to treat and potentially deadly. Disadvantaged populations such as the Indigenous people in remote tropical northern Australia have high burdens of *S*. *pyogenes* skin infection, and rheumatic heart disease. An effective vaccine would be a benefit, but none is approved for clinical use. We analysed data from 1810 *S*. *pyogenes* isolates from north Australia obtained over 28 years, to determine the potential of a previously described *S*. *pyogenes* vaccine candidate to be effective in this region. Only one quarter of the isolates from skin infections had a surface antigen corresponding to any one of the 30 antigen variants in the candidate vaccine. Previous work in animals indicates potential cross-protection from the vaccine against strains with mismatched antigens. However, even if this occurs in humans, protection against skin infection strains would likely remain compromised, unless there were additional components in the vaccine. Further studies on cross-protection are critical to defining the potential of this type of vaccine in populations burdened with *S*. *pyogenes* skin infections and rheumatic heart disease.

## Introduction

The Indigenous population of the Australian Northern Territory is disproportionately impacted by *Streptococcus pyogenes*. There is a high prevalence and incidence of skin infection (pyoderma, impetigo, skin sores) in children, for which *S*. *pyogenes* is the primary cause [1–3]. The prevalence of rheumatic heart disease (RHD) is one of the highest in the world [4–6], and there is evidence indicating a relationship to skin infections as well as pharyngitis [7, 8]. Also, the incidence of invasive *S*. *pyogenes* infections is substantially higher than in the Australian non-Aboriginal population. [9–11]. The Northern Territory Aboriginal population would benefit greatly from an effective GAS vaccine.

Analysis of *S*. *pyogenes* isolates from the Northern Territory in a series of studies has provided a consistent picture of high genetic diversity, with coexistence of multiple strains, even in small communities [12–15]. This is similar to observations in other disadvantaged populations [16–21]. There is currently no evidence that fundamentally novel lineages arise in the Northern Territory, so a current model is that social determinants leading to high skin infection prevalence facilitate on-going sustainable transmission of numerous lineages that ultimately originated outside the Northern Territory [22].

*S*. *pyogenes emm* types differ in their associations with anatomical sites. This has been described using the well-established concept of “*emm* patterns” [23, 24], with pattern A-C being throat associated, pattern D skin associated, and pattern E not associated with clear tropism. Previous work in the Northern Territory has shown such associations between *emm* pattern and site of isolation [14, 25].

More recently, the “*emm* cluster” scheme for classification of M protein variants was developed [21, 24, 26]. This is based on full length M protein sequences, and there is demonstrated correlation between *emm* cluster and host biomolecule binding specificity. The *emm* cluster nomenclature is an elaboration and modification of the *emm* pattern scheme. Phylogenetic analysis of the whole M protein sequence distinguished two main clades, X and Y. In Clade X, *emm* clusters E1-E6 are approximately equivalent to *emm* pattern E. In Clade Y, *emm*-clusters D1-D5 (D1-5) are approximately equivalent to *emm* pattern D, and *emm* clusters A-C1-A-C5 (A-C1-5) are approximately equivalent to *emm* pattern A-C. Also, some *emm* types within the X and Y clades are not related closely enough to other sequences to be assigned to clusters. Here, we term these collectively as “Clade Y/X. Other variants outside the X and Y clades are classified as “Outlier”.

*S*. *pyogenes* vaccine development strategies have largely been based on the M protein. A dominant hypothesis has been that immunity is *emm* type specific. However, it has been shown that immune sera from rabbits immunised with a 30-valent M-protein-based vaccine candidate (30mer vaccine) had significant killing activity against a large percentage of a test set of *S*. *pyogenes* strains with non-vaccine *emm* types [27, 28]. Furthermore, cross protection is more likely to occur within *emm* clusters [26]. Evidence for immunity across *emm* types, in particular within *emm* clusters, has been shown to arise from *S*. *pyogenes* impetigo infections in humans [29].

Vaccine candidates targeting conserved regions of the M protein rather than the highly variable N-terminal component have been developed. [30, 31]. These are primarily based upon peptides derived from the conserved C-repeat region. An extensively studied example is the C-region derived J8 B-cell epitope fused to non-streptococcal peptides and conjugated to diphtheria toxoid. This approach has reached human trials [32].

We aimed to use an archive of Northern Territory *S*. *pyogenes* isolates of known *emm* type, with corresponding data on site of isolation, epidemiology and clinical features, to understand the Northern Territory *S*. *pyogenes* population structure. Specifically, we sought to classify types according to the *emm* cluster classification scheme and anatomical site of isolation, to estimate the effectiveness of the candidate 30mer vaccine in this population. The long time-span over which isolates were collected (1987–2015) provided insight into strain persistence, total diversity, and diversity at time points. We identify knowledge gaps that could be prioritised in future studies directed towards the implementation of *S*. *pyogenes* vaccination in the Northern Territory.

## Methods

This study utilises data from stored *S*. *pyogenes* isolates, derived from hospital and community-based settings in the Northern Territory. *Emm* typing data and clinical information were available for 1810 of the isolates in the collection (S1 Dataset). These comprised 1713 isolates analysed as part of a study of GAS *emm* diversity in the Northern Territory covering 1987–2008 (Towers et al, 2013), which included isolates from studies described in a number of publications [7, 8, 12–14, 25, 33–37]. In general, these isolates were from remote dwelling community members through testing for throat carriage, or investigating clinical syndromes consistent with *S*. *pyogenes* infection. Eighty-two isolates were *emm* typed as part of a study of invasive GAS infections at two Northern Territory hospitals from 2011–2013 [10] and a further 15 isolates were collected and *emm* typed as part of a response to a cluster of cases of acute rheumatic fever (ARF) in 2014–5 [38].

Data extracted for each isolate were *emm* type, date of isolation (with the exception of one isolate for which date was unavailable), anatomical site of isolation, and disease description. Isolates derived from the throat that had a disease description of “pharyngitis” were classified as “pharyngitis” isolates. Other throat derived isolates were classified as “throat carriage”. Isolates from the skin that had a collection site of “skin sore/abscess” were classified as “skin and soft tissue infections” (SSTI). These encompassed disease descriptions including pyodermas (skin sores), abscesses, cellulitis, necrotising fasciitis, and infected burns, with the majority being pyodermas. All other skin isolates had a collection site recorded as “normal skin”. Forty-one isolates were from other anatomical sites such as the urogenital tract or the ear. They were classified as “other”. Isolates in the “other” category were omitted from determination of correlation between *emm* cluster and anatomical site because of the low probability of useful correlations being observed. The complete data set is provided (S1 Dataset).

The *emm* type for each isolate was assigned to an *emm* cluster in accordance with the conversion provided by the USA Centres for Disease Control, at https://www.cdc.gov/streplab/downloads/distribution-emm-types.pdf. The *emm* type nomenclature was updated according to the conversion provided by the USA Centres for Disease Control at https://www.cdc.gov/streplab/downloads/emm-numeric-sort.pdf.

Categorisation of *emm* types according to predicted protective efficacy of the 30mer vaccine was on the basis of cross opsonisation experiments in animals [27, 28]. *Emm* types encompassed in the 30mer vaccine were classed as “vaccine”. Non-vaccine *emm* types that were more than 50% killed in both cross opsonisation studies [27, 28] were classed as “cross opsonisation-positive”. Non-vaccine *emm* types that were more than 50% killed in one of the two cross opsonisation studies were classed as “cross opsonisation-equivocal”. Non-vaccine *emm* types that were less than 50% killed in both of the crossopsonisation studies were classed as “cross opsonisation-negative”. Non-vaccine *emm* types that were untested in the cross opsonisation experiments were classed as “cross opsonisation unknown”. This convention is similar to that described by Williamson and co-workers [19].

For some analyses, all *emm*55 isolates were excluded. This is because 65 of the 81 *emm*55 isolates were isolated in 2005, coinciding with a large outbreak of acute post-streptococcal glomerulonephritis (APSGN) [39]. All remaining *emm*55 isolates were isolated in 1991 (14 isolates) or 1992 (one isolate). This indicates that *emm*55 causes outbreaks, which has the potential to bias analyses.

Confidence intervals for proportions were calculated using the Wilson variant of the binomial proportions method, without continuity correction, at this site: http://vassarstats.net/prop1.html. The significance of differences in proportions were assessed using the Chi squared N-1 test, accessed at https://www.medcalc.org/calc/comparison_of_proportions. p values stated in the text are modified by application of the Bonferroni correction for multiple testing.

The Simpsons Index of Diversity (*D)* was calculated as described by Hunter and Gaston [40], using a Microsoft Excel application developed by the authors.

This study was classed by the Human Research Ethics Committee of the Northern Territory Government Department of Health, and the Menzies School of Health Research as meeting the requirements of a negligible risk activity, and eligible for waiver of full ethical review. This is documented in a letter to P.M.G on August 30, 2018.

## Results

The 1810 *S*. *pyogenes* isolates collected between 1 January 1987 and 26 February 2015 were classified into *emm* clusters (Table 1).

**Table 1:** Distribution of the 1810 isolates in this study into *emm* clusters.

*Emm* cluster	Number of isolates	% total	Group % total
AC-1	1	0.06	4.81
AC-2	14	0.77	
AC-3	42	2.32	
AC-4	25	1.38	
AC-5	5	0.28	
Clade_X_4	1	0.06	7.57
Clade_Y_2	1	0.06	
Clade_Y_3	54	3.0	
Clade_Y_5	9	0.50	
Clade_Y_6	13	0.72	
Clade_Y_8	2	0.11	
Clade_Y_14	18	0.99	
Clade_Y_15	21	1.16	
Clade_Y_16	9	0.50	
Clade_Y_17	2	0.11	
Clade_Y_20	4	0.22	
Clade_Y_21	3	0.17	
D1	45	2.49	33.11
D2	88	4.86	
D3	26	1.44	
D4	416	22.99	
D5	24	1.33	
E1	83	4.59	47.73
E2	106	5.86	
E3	262	14.48	
E4	169	9.33	
E5	1	0.06	
E6	243	13.42	
Outlier 1	81	4.48	5.75
Outlier 2	4	0.22	
Outlier 5	14	0.77	
Outlier 6	5	0.28	
Unknown	19	1.10	1.05

The distribution of the *emm* types identified in the study into *emm* clusters is shown in Table 2. One hundred and one *emm* types were identified, encompassing all defined *emm* clusters.

**Table 2:** Distribution of the *emm* types identified in this study into 30mer vaccine protection categories.

*Emm*-cluster	Vaccine	Cross opsonisation-positive	Cross opsonisation-equivocal	Cross opsonisation-negative	Cross opsonisation-unknown
A-C1					142
A-C2					197
A-C3	1				1–4
A-C4	12				39, 193, 229
A-C5	3				
					
Clade_X					236
					
Clade_Y	6, 14, 18, 19, 24	74, 105, 122			57, 218, 233
					
D1				54	207
D2			71, 100		
D3		123			217
D4		33, 52	53	70, 80, 116	41, 56, 86, 91, 93, 98, 101, 108, 178, 192, 225, 230
D5		97			
					
E1	4, 78	60			165
E2	92	68, 76			13, 90, 106, 110, 117, 166
E3	44, 49, 58, 82, 87	15, 25, 183			9, 103, 231, 144
E4	2, 22, 28, 73, 77, 89, 114	8, 102, 109		124	88, 112, 232
E5					170
E6	11, 75, 81	48, 65, 69, 85	63		67, 99
					
Outlier			95	55	241, 222
					
Unknown					stG653, stGrobn
					
Total *emm* types	26	20	5	6	45

Variants of *emm* clusters D1-5 and E1-6 are numerically dominant, together accounting for 80.8% of the isolates. The majority (69.4%) of *emm* cluster D1-5 isolates are *emm* cluster D4, with these isolates comprising nearly one quarter of the entire collection and encompassing 18 *emm* types.

**Fig 1:**
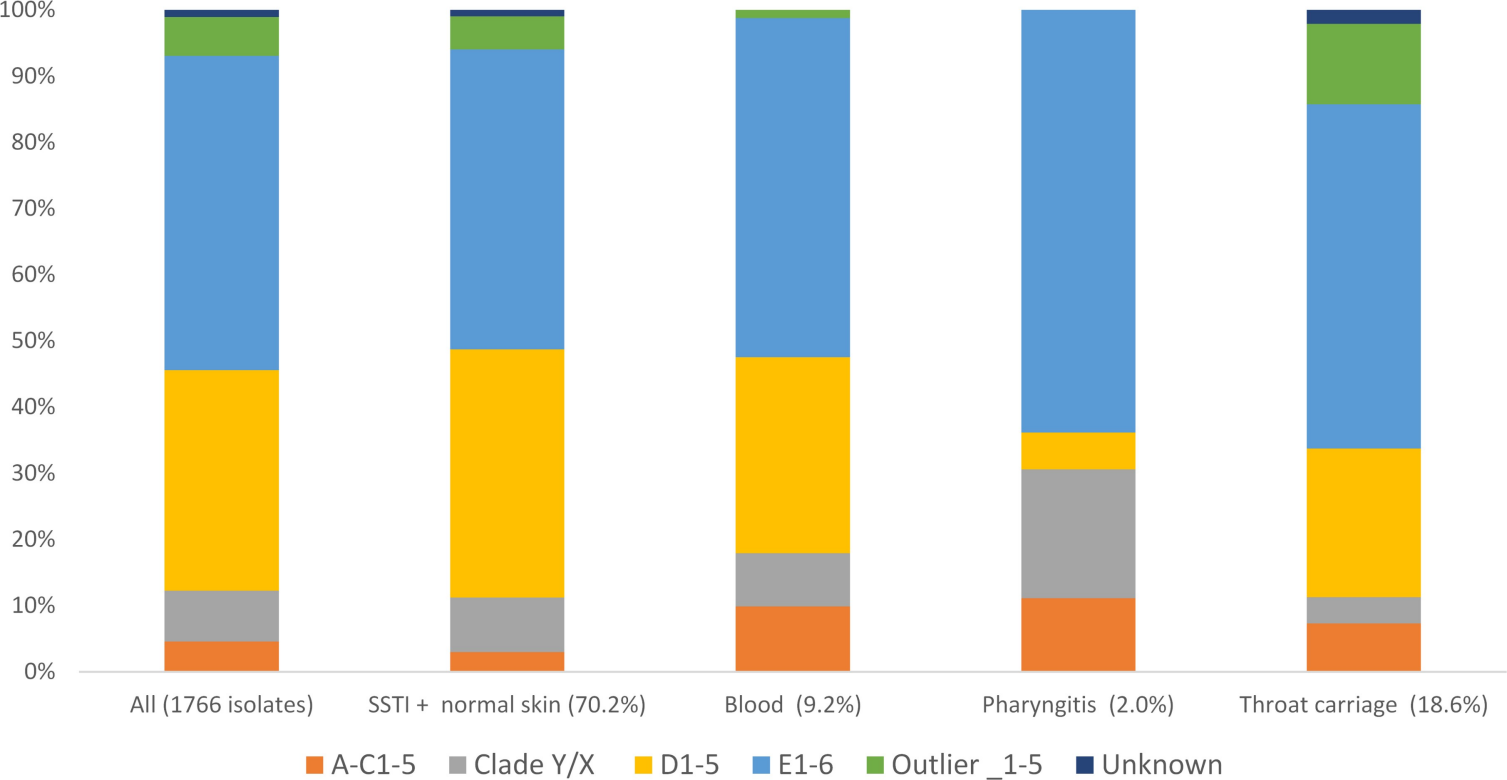
Distribution of isolates from different anatomical sites into *emm* clusters.

To reveal anatomic tropism, the relationship between *emm* cluster and anatomical site of isolation was determined (Fig 1, S2 Dataset). For this analysis, 41 isolates from anatomical locations in the “other” category for site of isolation were excluded (see Methods). Strongest statistical support (p<0.007)) was for enrichment (i.e. an elevated proportion) of *emm* cluster D1-5 isolates in skin isolates with respect to throat carriage isolates, enrichment of *emm* cluster A-C1-5 isolates in blood with respect to SSTI, and enrichment of cluster Outlier_1–5 (including *emm*55 isolates) in throat carriage with respect to blood. Other statistically significant but less strongly supported associations included enrichment of *emm* cluster D1-5 isolates in SSTI with respect to pharyngitis (p = 0.007), and *emm* cluster A-C1-5 in throat carriage with respect to SSTI (p = 0.028).

**Fig 2:**
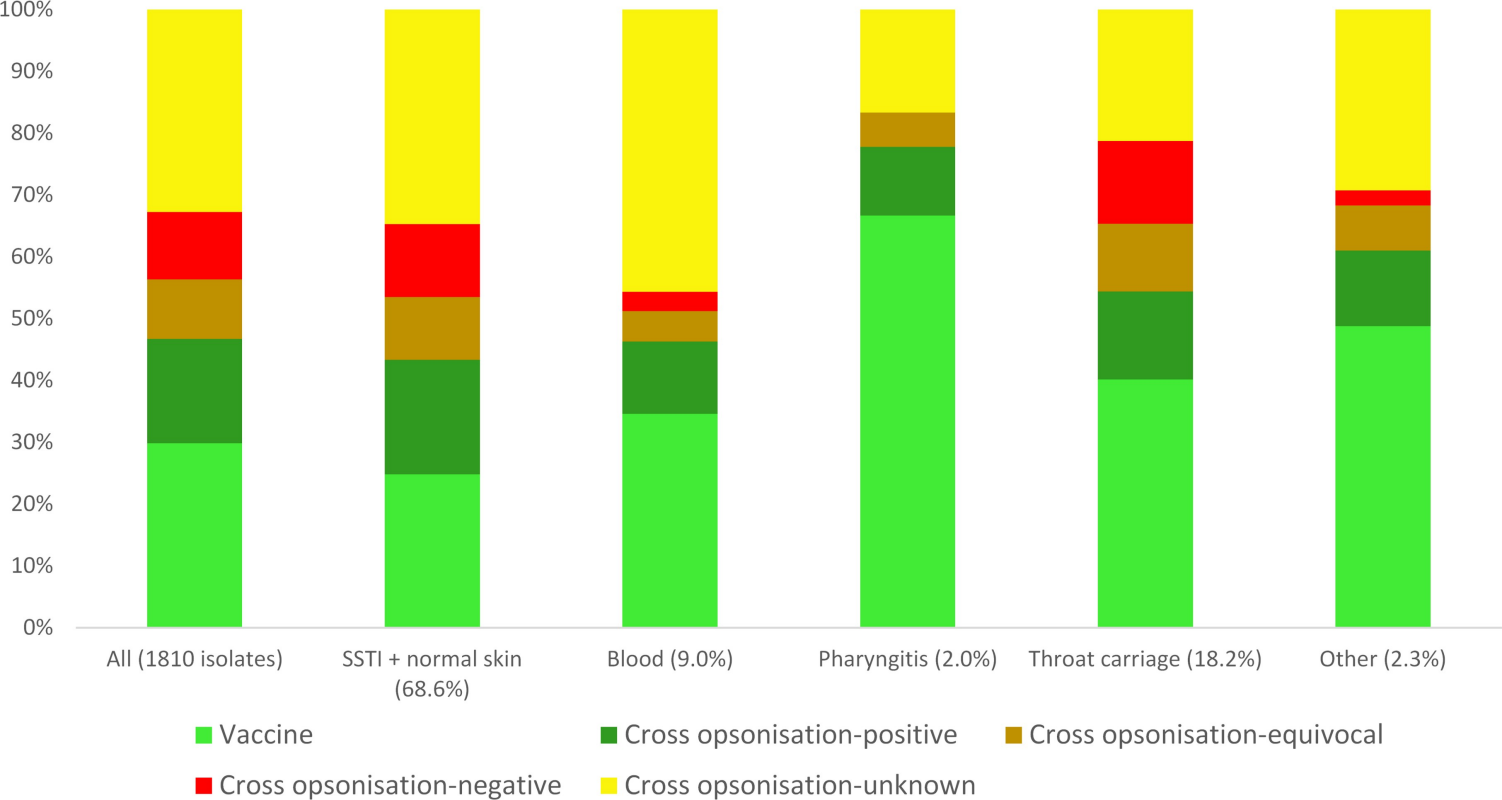
Distribution of isolates from different anatomical sites amongst vaccine protection classes.

The distribution amongst vaccine classes was determined (Fig 2, S3 Dataset). Strongest statistical support (p<0.008) was for enrichment of vaccine *emm* type isolates in pharyngitis and throat carriage with respect to SSTI, enrichment of vaccine *emm* type isolates in pharyngitis with respect to normal skin, and enrichment of cross opsonisation-unknown isolates in SSTI with respect to throat carriage and also in blood with respect to throat carriage. If *emm*55 isolates are included, there was also strong statistical support for enrichment of cross opsonisation-negative *emm* type isolates in throat carriage with respect to SSTI, and in throat carriage with respect to “other”. Other less strongly supported differences can be seen in Part B of S3 Dataset. The numerically abundant *emm* cluster D4 isolates were specifically examined, because of their likely significance to vaccine implementation in the study area. Notably, there are 264 isolates of *emm* types that are *emm* cluster D4 and cross opsonisation-unknown. This is 14.6% of the entire collection and 44.5% of the non-vaccine, cross opsonisation-unknown *emm* type isolates.

We then determined the diversity of the isolates on the basis of *emm* type, as high diversity, particularly of non-vaccine *emm* type strains, may complicate vaccine development. Diversity was calculated for each combination of vaccine protection class and anatomical site of isolation (Table 3).

**Table 3:** Diversities of isolates in different protection classes and anatomical sites.

		Vaccine	Cross opsonisation-positive	Cross opsonisation-equivocal	Cross opsonisation-negative	Cross opsonisation-negative, *emm*55 excluded	Cross opsonisation-unknown
	Total *emm* type number	26	19	6	6	5	50
**SSTI**	Numbers of *emm* types	21	19	6	6	5	38
	*D*	0.93	0.93	0.78	0.82	0.80	0.94
**Blood**	Numbers of *emm* types	19	12	4	4	3	25
	*D*	0.90	0.95	0.65	0.90	0.83	0.93
**Pharyngitis**	Numbers of *emm* types	13	4	2	0	0	6
	*D*	0.95	1.0	1.0	-	-	1.0
**Throat carriage**	Numbers of *emm* types	18	13	5	4	3	20
	*D*	0.90	0.89	0.73	0.22	0.80	0.92
**Normal skin**	Numbers of *emm* types	3	3	2	3	3	7
	*D*	1.0	1.0	0.67	0.6	0.6	0.81
**Other**	Numbers of *emm* types	15	4	3	1	1	11
	*D*	0.97	0.90	1	-	-	0.99

It was notable that the isolates characterised as “cross opsonisation-unknown” were particularly diverse. Diversity was also determined for isolates obtained in calendar years that yielded >100 isolates (1991, 95, 97, 99, 2004, 05). The *D* values were 0.94 (1999, 2004, and 2005), 0.95 (1991 and 1995) and 0.96 (1997), showing that high diversity is not a consequence of the long period over which isolates were collected.

**Fig 3:**
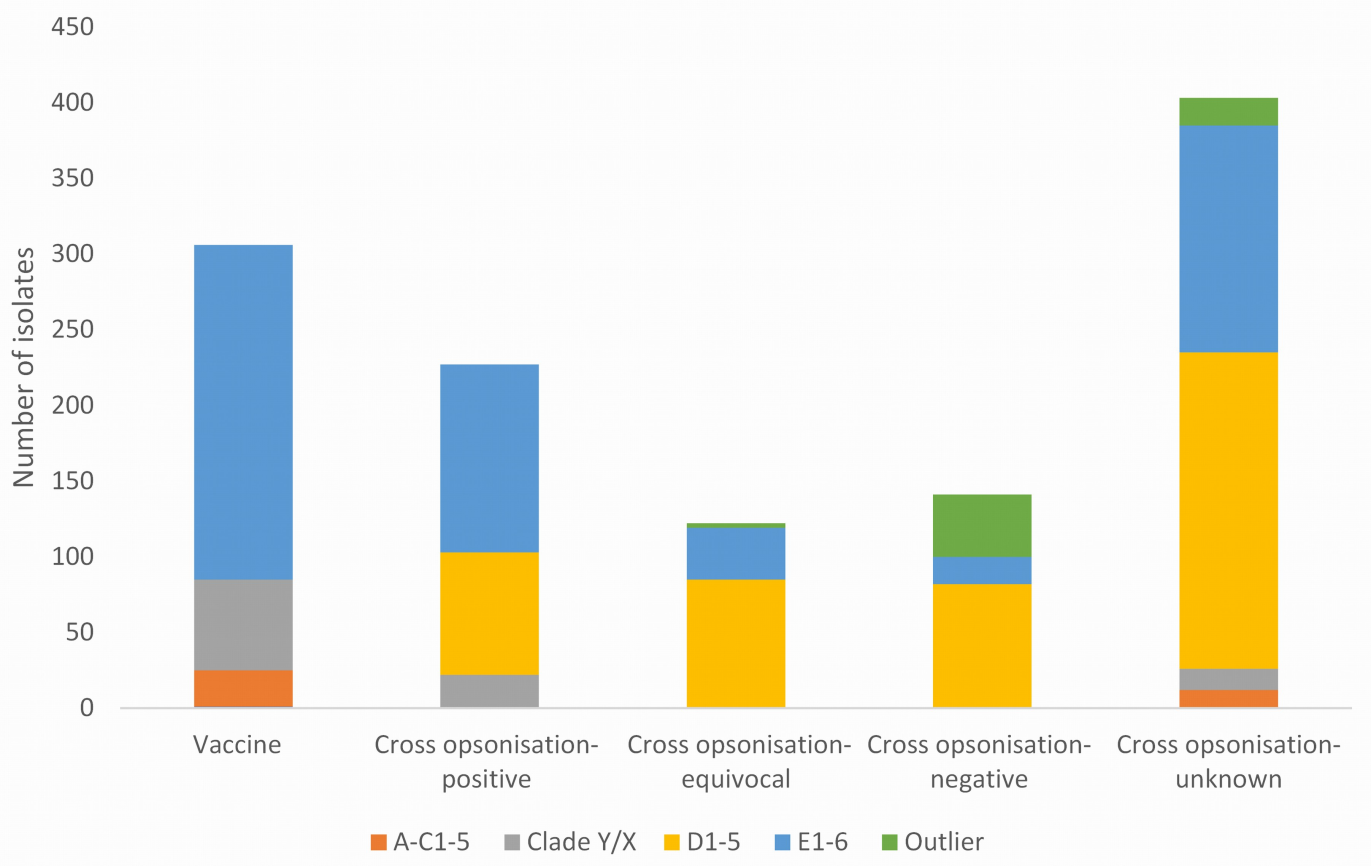
Distribution of SSTI isolates according to *emm* cluster and vaccine class.

To further investigate the SSTI isolates, their distribution into vaccine coverage classes and *emm* clusters was determined (Fig 3, S4 Dataset). Fig 3 shows large differences between the vaccine protection classes with respect to *emm* cluster make-up, and the S4 Dataset shows that a large proportion of these differences are strongly supported by statistical analysis (p <0.006).

**Fig 4:**
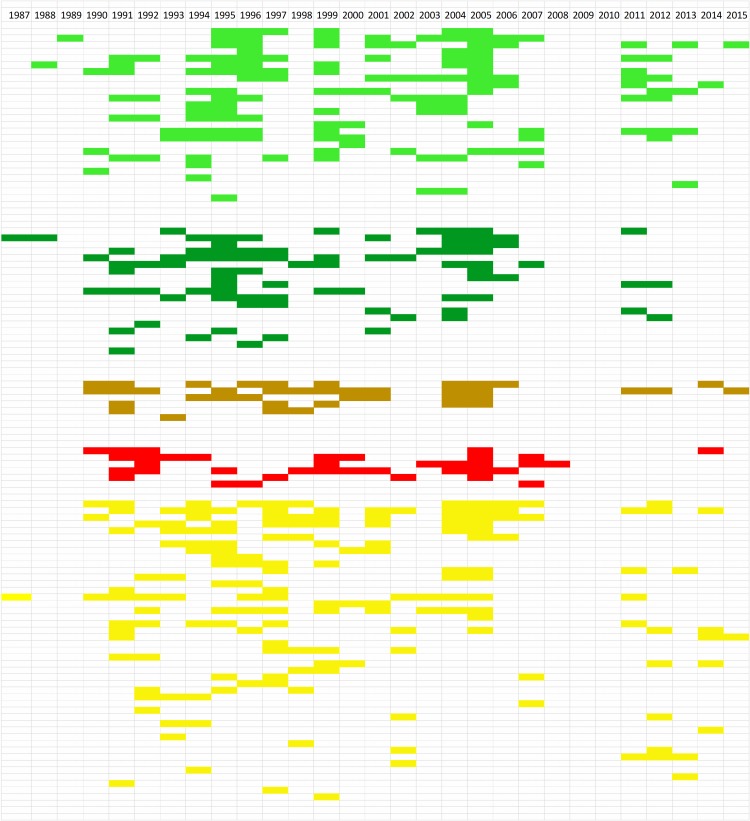
Persistence of *emm* types. Each row with colours blocks is an *emm* type. The top row is calendar year. A block is coloured if there was at least one of that *emm* type isolated in that year. Pale green: vaccine types; dark green: cross opsonisation-positive, brown: cross opsonisation-equivocal; red: cross opsonisation-negative; yellow: cross opsonisation-unknown. The Excel file from which this is derived, containing *emm* type numerical information, is provided as S5 Dataset.

**Fig 5:**
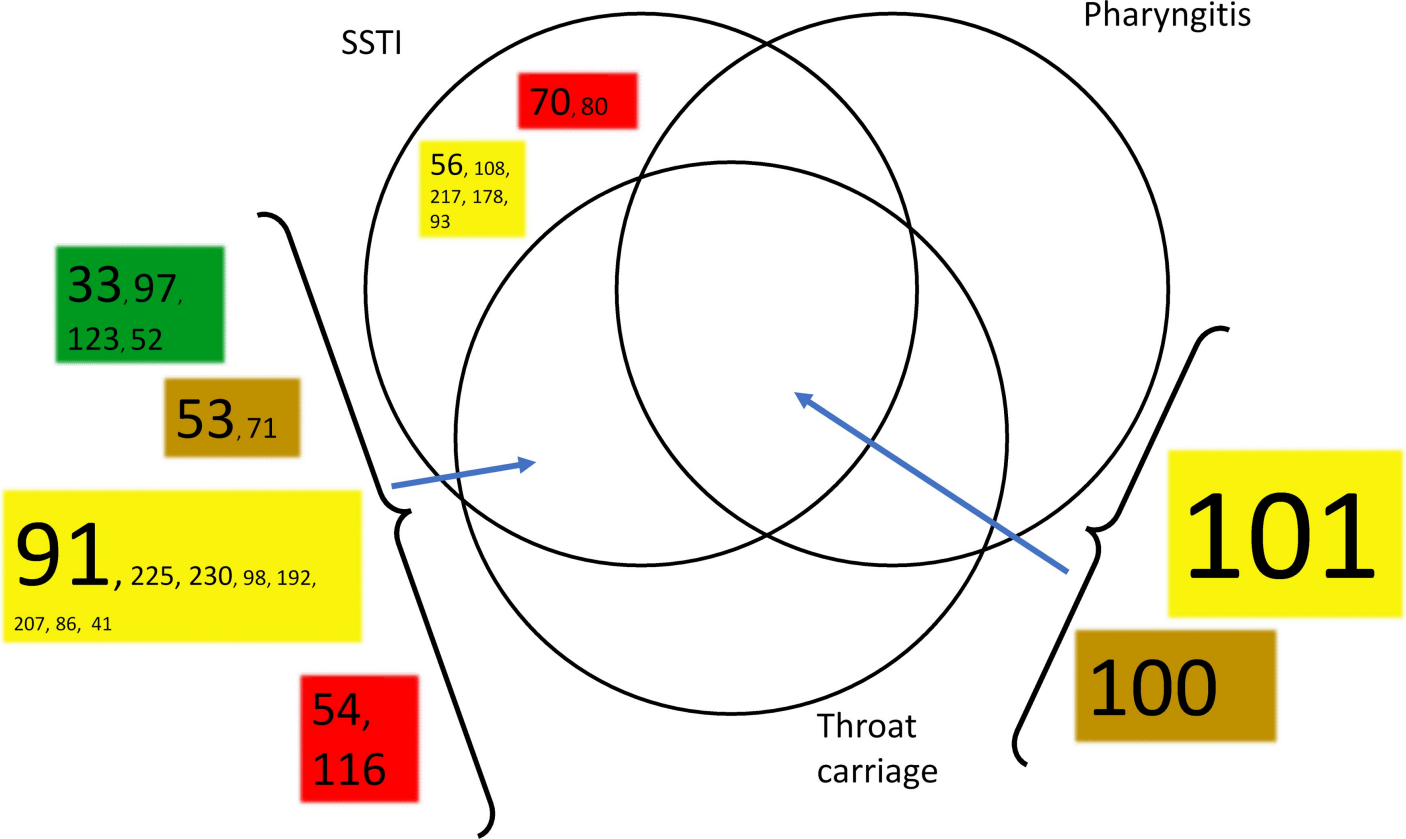
Relationship between *emm* types within *emm* cluster D1-5, number of isolates, vaccine class, and anatomical sites in which *emm* types were identified. The font size is proportional to the number of isolates from SSTI infections, where the number is >9. Pale green: vaccine types; dark green: cross-opsonisation- positive, brown: cross opsonisation-equivocal; red: cross opsonisation-negative; yellow: cross opsonisation-unknown. B. *emm* cluster E1-6.

**Fig 6:**
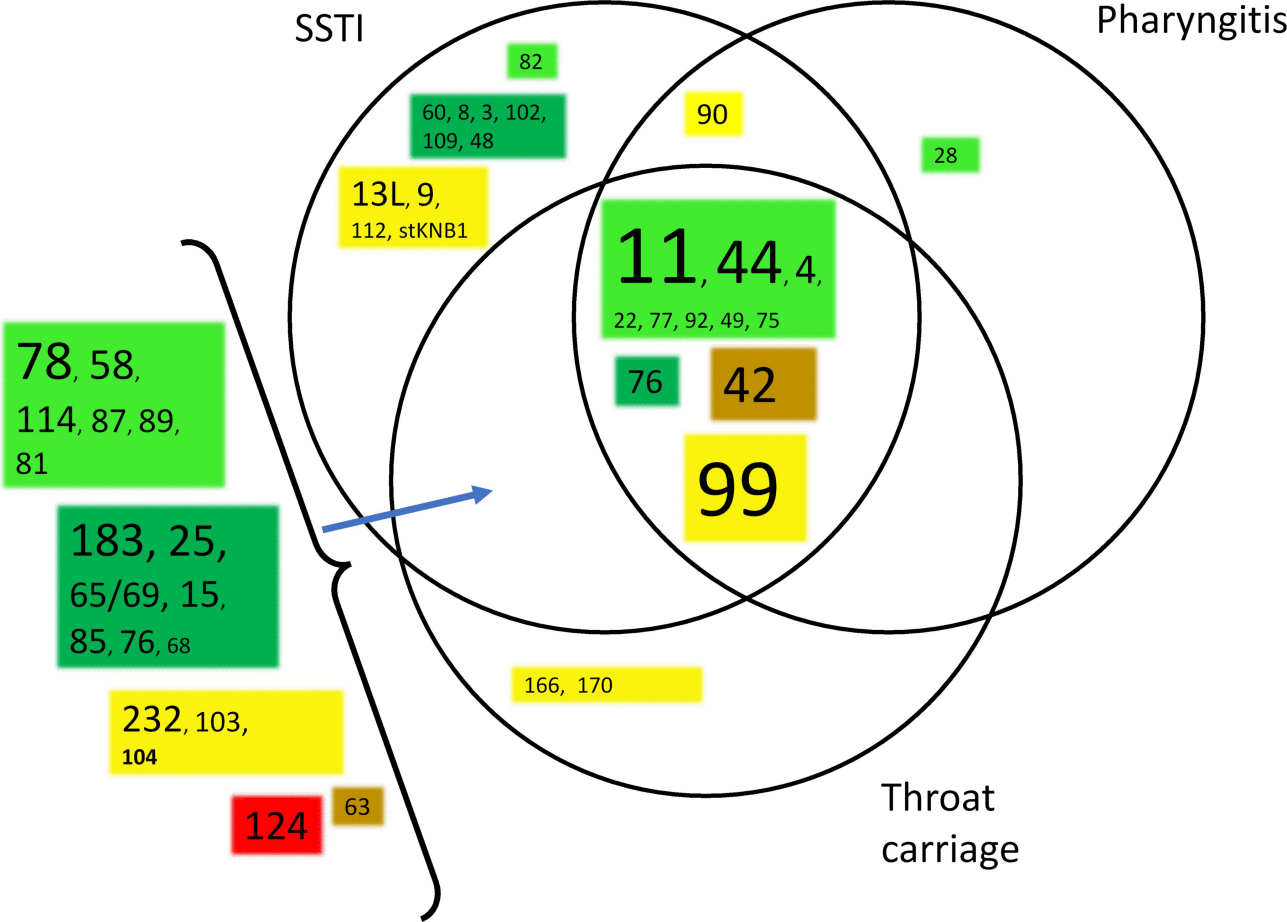
Relationship between *emm* types within *emm* cluster E1-6, number of isolates, vaccine class, and anatomical sites in which *emm* types were identified. The font size is proportional to the number of isolates from SSTI infections, where the number is >9. Pale green: vaccine types; dark green: cross-opsonisation- positive, brown: cross opsonisation-equivocal; red: cross opsonisation-negative; yellow: cross opsonisation-unknown.

*Emm* type persistence was determined on the basis of the number of calendar year(s) in which each *emm* type was isolated. This is shown graphically in (Fig 4, S5 Dataset), with the *emm* types resolved on the basis of 30mer vaccine protection class. This analysis showed extensive co-existence of *emm* types within single calendar years, in combination with numerous examples of long term *emm* type persistence. There was no evidence for correlation of persistence with vaccine class. The study data set encompasses many examples of the same *emm* types being isolated from different anatomical sites. This is relevant to understanding strain tropism and mobility between anatomical sites. In combination with strain abundance data, this information can assist in prioritising *emm* types and *emm* clusters for further study. The relationship between number of isolates of each *emm* type, site of isolation, and *emm* cluster was determined. The results for the D1-5 and E1-6 *emm* clusters are shown in Fig 5 and Fig 6 and for the other *emm* clusters in S1 Fig. For the abundant *emm* clusters D1-5 and E1-6, there is extensive commonality in *emm* types isolated from different anatomical sites. In particular, this is seen with SSTI and throat carriage isolates. A similar analysis was performed for blood isolates (S2 Fig), and this showed that it was common for *emm* types found in blood to be isolated from different sites. For example, 40 of the 66 *emm* types (60%) were also isolated from SSTIs and throat carriage. Complete numeric *emm* type distributions according to anatomical sites of isolation, *emm* cluster, isolate numbers and year(s) of isolation are provided (S6 Dataset).

## Discussion

Here we have described *emm* type diversity of *S*. *pyogenes* collected over nearly three decades in the tropical north of Australia. We found that less than one third of the isolates overall, and 25.2% of the SSTI isolates, are cognate with the 30mer vaccine. In the absence of cross protection between *emm* types, 30mer vaccine efficacy would be likely to be poor in the Northern Territory.

The bacterial population was described in terms of the *emm* cluster classifications scheme, its relationship with anatomical tropism, and previously reported cross opsonisation data for a subset of known *emm* types [27, 28]. We show that isolates of *emm* types for which there are no cross-opsonisation data are abundant and diverse. This limits inference of vaccine efficacy and suggests directions for further work. The relationship between *emm* cluster and anatomical tropism was broadly consistent with previous reports. However, there were numerous instances of nominally skin tropic strains colonising the throat without causing signs or symptoms of pharyngitis.

The most abundant *emm* cluster categories in the study were E1-6 and D1-5. *Emm* cluster D1-5/*emm* pattern D strains are regarded as “skin tropic” [24, 26, 41] and our findings were consistent with this, with the proportions of cluster D1-5 in both pharyngitis and throat carriage isolates significantly less than for SSTI isolates (pharyngitis p = 0.007, throat carriage, p<0.007). *Emm* cluster/*emm* pattern E1-6 strains are regarded as non-tropic and consistent with this, no significant differences in relative prevalence at different anatomical sites were observed. Cluster A-C1-5 strains are regarded as throat tropic [24, 26]. The point estimates for the relative prevalence were higher for throat carriage and pharyngitis isolates than for non-throat isolates, but the difference was only significant for throat carriage isolates (p = 0.028), likely due to the larger number of throat carriage isolates in the study. The point estimate for the relative prevalence of Cluster A-C1-5 in blood isolates was similar to that for throat isolates and significantly higher than the relative proportion in SSTI (p<0.007). However, 12 of the 13 Cluster A-C1-5 isolates from blood were *emm*197 isolates isolated in connection with a cluster of invasive infections in 2012 [10], compromising the generalisability of this finding. The *emm* Cluster Clade Y/X isolates encompass a low frequency of throat carriage isolates as compared to pharyngitis isolates, and normal skin isolates. This is not currently understood. Given that Clade Y/X *emm* cluster category is composed of very diverse *emm* types, it is likely that the category encompasses a range of anatomical tropism conferring properties, making these results difficult to interpret. For the Outlier category, the only significant differences observed were for comparisons including *emm*55 isolates. Specifically, all the throat carriage isolates in the Outlier category were *emm*55 isolated in 2005, and likely associated with an outbreak of APSGN [39]. Therefore, the observation of elevated Outlier category *emm* types in throat carriage isolates may not be generalisable to the Northern Territory when there is not an *emm*55 outbreak in progress. In summary, tropism observed is either consistent with current models, explainable with sampling effects, or anomalous but involving a diverse *emm* cluster category.

The proportions of *emm* cluster categories derived from different anatomical sites were similar to findings from a meta-analyses of anatomical tropism on the basis of *emm* pattern [41]. However, previous analyses have not generally differentiated between pharyngitis and throat carriage isolates. Our study identifies a weaker tropism signal, on the basis of point estimates, for throat carriage strains, with many examples of the same *emm* types being recovered from SSTIs and throat carriage. This suggests that adaptation to throat carriage differs from adaptation to cause pharyngitis. Pharyngitis is rare in tropical Northern Territory, despite a high burden of skin disease and RHD [7, 42]. Thus, in this setting it appears that nominally skin tropic strains have markedly impaired capacity to cause symptomatic pharyngitis but do not experience strong barriers to colonising the throat. Open questions are the contributions of throat colonisation and SSTIs to ARF/RHD, and the extent to which throat colonisation contributes to sustainable transmission of SSTIs.

The coverage by the 30mer vaccine in the absence of cross protection is 66.7%, 40.1% and 25.2% for the pharyngitis, throat carriage and SSTI isolates respectively, with the reduced coverage of SSTI isolates in comparison to pharyngitis and throat carriage isolates strongly supported by statistical analysis (S3 Dataset) (p<0.008). The high coverage of the pharyngitis isolates is unsurprising given that the 30mer vaccine was designed with reference to strains circulating in North America, where pharyngitis is the dominant *S*. *pyogenes* infection [27]. The intermediate figure for throat carriage is consistent with our finding of weaker tropism for throat carriage, and extensive commonality of *emm* types from SSTI and throat carriage.

Isolates of *emm* types that have not been tested for cross reaction are numerous and diverse, with the proportion in SSTI isolates significantly higher than in throat carriage isolates (S3 Dataset). Extrapolation from cross opsonisation data could perhaps be used to attempt to predict 30mer vaccine efficacy. For example, from the top data row of S3 Dataset, 490 SSTI isolates are of non-vaccine *emm* types that have been tested for cross opsonisation. Of these, 349 (71.2%) are of cross opsonising *emm* types. A point estimate regarding vaccine efficacy can be made by assuming that the same proportion of isolates of “non-vaccine, cross opsonisation-unknown” *emm* types would show cross opsonisation. 71.2% of 415 isolates is 295 isolates. This generates an efficacy estimate regarding SSTI isolates of: (305 vaccine *emm* type isolates + 227 cross opsonising *emm* type isolates + 122 equivocal cross opsonising *emm* type isolates, + 295 (inferred) non-vaccine cross opsonisation-unknown isolates)/1210 total SSTI isolates = 78.4%. This could be regarded as promising. However, this estimate is potentially optimistic, because it encompasses an assumption that there will indeed be cross protection in humans against all the non-vaccine cross opsonising *emm* types. This remains to be demonstrated. Accordingly, while the above calculation may be useful in contributing to a conceptual framework for future research, we do not believe this defines a current robust evidence-based estimate for 30mer vaccine effectiveness in the Northern Territory.

The breakdown of the SSTI isolates on the basis of *emm* cluster and vaccine protection class, and the diversity determinations add detail to this picture (Fig 3, S4 Dataset). What is striking is the strong relationship between 30mer vaccine protection class and *emm* cluster composition. Significant differences in frequencies may be expected when comparing the “vaccine” class with other classes, because the vaccine contains no *emm* Cluster D1-5 antigens. However, we also observed strongly supported significant differences (p<0.008) regarding *emm* types that gave different cross opsonisation results i.e cross opsonisation-positive, -intermediate and -negative. It is noteworthy that the relative contributions of isolates that are *emm* Cluster E1-6 becomes less as the cross opsonisation results become more negative. The inverse is seen with *emm* cluster D1-5. This is consistent with correlation of *emm* cluster with cross protection [26], and indicates that the cross opsonisation data is biologically and potentially clinically meaningful. It was also noted that within the cross opsonisation-negative category there were four *emm* cluster D1-5 *emm* types (*emm*54, *emm*116, *emm*70 and *emm*80) and only a single *emm* cluster E1-6 *emm* type (*emm*124). This emphasises that *emm* cluster E1-6 isolates contribute very little to the cross opsonisation-negative isolates in terms of number or diversity. The substantial proportion of Outlier isolates in this category were *emm*55, many of which were associated with an APSGN outbreak [39]. Removing these from consideration emphasises the dominance of *emm* cluster D1-5 in the cross opsonisation-negative category.

The overall picture is that the *emm* cluster E1-6 isolates are overwhelmingly of *emm* types that are either cognate with the 30mer vaccine, tested positive in cross opsonisation assays, or are of *emm* types untested for cross opsonisation. In contrast, the *emm* cluster D1-5 isolates (which are primarily *emm* cluster D4), are all non-cognate with the 30mer vaccine, and are less likely to be of *emm* types that tested positive in cross opsonisation assays. Also, just over half the isolates of cross opsonisation-unknown *emm* types are *emm* cluster D1-5. The other *emm* cluster categories are of less concern. In particular there are no *emm* cluster A-C1-5 or Clade Y/X isolates in the cross opsonisation equivocal or negative categories, and very few that are untested. Outlier isolates are either cross opsonisation-negative (APSGN associated *emm*55) or a small number of isolates of untested *emm* types. In summary, these results lead to the prediction that *emm* clusters D1-5, in particular *emm* cluster D4, are particularly problematic with respect to 30mer vaccine efficacy against SSTIs in the Northern Territory. It is relevant that Frost and co-workers found evidence that eliciting an immune response against *emm* cluster D4 strains with a non-cognate *emm* type is inherently difficult [29].

The “blood” isolates in this study represent isolates from invasive infections, and so are of inherent interest. These are diverse, with the 162 blood isolates encompassing 66 *emm* types, 57 of which have been found in this study at other anatomical sites. The distribution between *emm* clusters and vaccine protection categories are broadly similar to SSTI isolates. *Emm*81 is the most abundant *emm* type in the blood isolates (*emm* cluster E6, 14 isolates, 8.6% of total). *Emm*81 is a “vaccine” *emm* type and was isolated in nine separate years, indicating persistence rather than particularly high prevalence at any time point. *Emm*81 has been associated with invasive infections in several studies outside Australia [43–45]. Of the nine *emm* types found only in blood, seven are represented by only 1–2 isolates. Of greater interest are the remaining two *emm* types, *emm*197 (*emm* cluster A-C2) (12 isolates) and *emm*113 (E3) (10 isolates). Other studies have not identified these as dominant invasive strains, but *emm*197 has been identified in New Zealand associated with ARF [46] and *emm*113 has been classified as “invasive”[47]. Both are non-vaccine *emm* types with no cross opsonisation information, and in the Northern Territory were isolated in the same geographical region in 2012 (*emm*197) and 2011, 2012, 2013 (*emm*113), suggesting outbreaks [10]. These *emm* types are candidates for future study and surveillance.

Isolates from sites other than the skin, throat or blood are highly diverse (S1 Dataset) with the 41 isolates encompassing 34 *emm* types, and no *emm* types are represented by more than two isolates. This is unsurprising giving the diversity of sites and the long period over which isolates were collected.

It is likely that the 30mer vaccine will not provide good protection against APSGN in the NT. Of three APSGN outbreaks in the Northern Territory, *emm*55 *S*. *pyogenes* is associated with the largest, in 2005 [39]. *Emm*55 is non-cognate with the vaccine and cross opsonisation-negative. A recent review indicates that poor 30mer vaccine coverage against APSGN associated strains is predicted in Australia, and also in South America, where *emm*55 appears to be an important cause of APSGN outbreaks [48]. Inclusion of an *emm55* antigen in any *S*. *pyogenes* vaccine targeted to remote regions with disadvantaged populations may be justifiable.

The population structure of *S*. *pyogenes* in the Northern Territory has now been clearly established as encompassing high diversity and long term *emm* type persistence, resulting in high diversity at any time point and a high proportion of cluster D1-5 and E1-6 strains and isolates. This is similar to other studies based on populations with high burdens of skin infections and ARF/RHD [16–18, 20]. In particular the picture is remarkably similar to what was observed in New Zealand by Williamson and co-workers [19], where high *emm* type diversity, correlation of ARF burden with pyoderma burden and cluster D1-5 prevalence, and poor 30mer vaccine coverage of skin-derived isolates, were all observed.

This analysis defines specific *emm* types that could be prioritised for further study, in addition to the blood-associated invasive *emm* types mentioned above. For example, *Emm* cluster D1-5 types, for which there are no cross opsonisation data, and are relatively abundant and persistent in this study setting were *emm*101 (*emm* cluster D4), *emm*91 (D4), and *emm*56 (D4). *Emm*101 and *emm*91 were also of high relative prevalence in pyoderma isolates from New Zealand [19]. S6 Dataset is a potentially useful resource for prioritising further work.

The major limitation of this study is that the isolates were not collected systematically but were a convenience sample. However, the high diversity at multiple time points, in combination with evidence for extensive strain persistence, suggests that the isolates do provide a useful picture of *S*. *pyogenes* diversity in the Northern Territory over a period of decades. Another limitation is the small number of pharyngitis isolates, although this is in large part a consequence of the low prevalence of pharyngitis in the study area.

We conclude that the effectiveness of the 30mer vaccine in the study area would be dependent on *emm* type cross protection in humans, which cannot currently be estimated with any certainty. If there is cross protection in accordance with cross opsonisation data, then the 30mer vaccine can be reasonably predicted to provide good protection against all *emm* clusters except D1-5, and possibly Outlier, which together comprise 53.5% of the sample. Therefore, a potential strategy would be to combine the 30mer vaccine with a vaccine(s) specifically targeting *emm* cluster D1-5, and possibly *emm*55. It is of interest that a 26-valent precursor of the 30mer vaccine contains the *emm*33 and *emm*101 antigens [49]. These are both *emm* cluster D4. Both *emm* types are relatively abundant in our collection, with *emm*101 being the most abundant *emm* type (74 isolates), and *emm*33,represented by 32 isolates. Therefore, testing the 26mer vaccine, or a combination of the 30mer and 26mer, in the Northern Territory may be worth consideration. However, in the absence of cross protection, obtaining useful efficacy in the Northern Territory using an *emm* type specific vaccine may be difficult to achieve, in particular because coverage of the numerous *emm* Cluster E1-6 strains will be less, and obtaining coverage of the numerous and diverse *emm* cluster D1-5 may be impractical. Accordingly, determination of the correspondence between cross opsonisation data and clinical efficacy is critical for defining future directions.

## Supporting information

S1 Checklist STROBE statement. The checklist used is that designed for observational studies.
(DOCX)

S1 Dataset Complete isolate by isolate dataset. (XLSX)

S2 Dataset Distribution of isolates on the basis of anatomical site of origin. **Part A:** Distribution of isolates on the basis of anatomical site of origin. These values were used to construct Fig 1. In Fig 1, the numbers from SSTI and normal skin were combined. 95% CI intervals were calculated, without correction for multiple testing (see Methods). No 95% CI intervals were calculated for the proportions in the bottom row, as these are in large part a reflection of sampling activity, rather than any biological properties of the isolates. **Part B:** Results of N-1 Chi-suared tests. This experiment addressed differences between isolates from different anatomical sites regarding their distribution into *emm* clusters. For example, the top left data square is derived from an N-1 Chi-squared test on the percentage of SSTI isolates that are *emm* cluster A-C1-5 (3% of 1210 isolates), compared with the percentage of blood isolates that are *emm* cluster A-C1-5 9.9% of 162 isolates). Because of potential for bias, the analysis was also performed with *emm*55 isolates omitted (starred data points) (see text). We took a conservative approach to assessing the credibility of the differences in proportions. We used the 70 cells in the table to apply a Bonferroni correction. The usual cut-off for significance is P = 0.05. 0.05/70 = 0.00071. Cells coloured red have values <0.0001, which equates to a Bonferroni corrected p value of <0.007, which we regard as strongly supporting the significance of the difference in proportion. Cells coloured orange have p values from 0.0001–0.0007, which equates to Bonferroni corrected p values of 0.007–0.05. We regard this as significant, but less strongly supporting the difference in proportion. The orientations of significant differences are shown using single letters reflecting the first letters of the anatomical sites. A “-”symbol designates where the comparison is of two 0% values.
(DOCX)

S3 Dataset Distribution of all isolates included in this study into 30mer vaccine classes. **Part A.** Distribution of the 1810 isolates included in this study into 30mer vaccine protection classes. These data were used to assemble Fig 2. Starred figures are with *emm*55 isolates omitted. 95% CI’s (see Methods) are provided for the distributions of isolates from different anatomical sites across 30mer protection classes. 95% CI’s are not provided for the distribution of isolates of different 30mer protection classes across sites of isolation, as this is impacted greatly by specimen collection activity, compromising the value of statistical analysis. **Part B.** This experiment addressed differences between isolates from different anatomical sites regarding their distribution into 30mer vaccine protection classes. For example, the top left data square is derived from an N-1 Chi-squared test on the percentage of SSTI isolates that are 30mer vaccine *emm* types (25.2% of 1210), vs the percentage of blood isolates that are 30mer vaccine emm types (34.6% of 162 isolates). Starred numbers were calculated with *emm*55 isolates omitted. As with S2 Data Set, a Bonferroni correction was applied. In this instance, 76 tests for significance were performed. Cells coloured red have values <0.0001, which equates to a Bonferroni corrected P -value of <0.0076, which we regard as strongly supporting significance. Cells coloured orange have P values from 0.0001–0.0006, which equates to Bonferroni corrected P values of 0.008–0.05. We regard this as significant, but less strongly supporting the difference in proportion. Starred values are with *emm*55 isolates omitted. The orientations of significant differences are indicated in the data cells, with the single letters representing the first letters of site of isolation.
(DOCX)

S4 Dataset Relationship between 30mer vaccine protection class and emm cluster for SSTI isolates. **Part A.** Relationship between 30mer vaccine protection class and *emm* cluster for SSTI isolates. Starred numbers are with emm55 isolates omitted. 95% CI’s are included for the percent values calculated with reference to the “vertical totals”; the number of isolates in each 30mer vaccine protection class. These are the values used for the analysis in Part B below, and also which were visualised in Fig 3. The percent values with reference to anatomical site (horizontal totals) will be in large part a function of specimen collection activity, so have not been subjected to statistical analysis. **Part B.** Results of N-1 Chi-squared tests. This experiment addressed differences between SSTI isolates of different *emm* cluster categories their distribution into 30mer vaccine protection classes. For example, the top left data square is derived from an N-1 Chi-squared test on the percentage of “vaccine *emm* type” isolates that are also “*emm* cluster A-C1-5” isolates (7.9% of 305 isolates) vs percentage of “cross-opsonisation positive” isolates that are also “*emm* cluster A-C1-5” isolates (0% of 227 isolates). Starred numbers were calculated with *emm*55 isolates omitted. As with S2 Data Set and S3 Data Set, a Bonferroni correction was applied. In this instance, 59 tests for significance were performed. Cells coloured red have p values <0.0001, which equates to a Bonferroni corrected P -value of <0.006, which we regard as strongly supporting significance. Cells coloured orange have P values from 0.0001–0.0008, which equates to Bonferroni corrected p values of ~0.006–0.05. We regard this as significant, but less strongly supporting the difference in proportion. Starred values are with *emm*55 isolates omitted. The orientations of significant differences are indicated in the data cells, with the single letters either designating “vaccine” (v), or the first letter of the cross-opsonisation descriptor. A “-”symbol represents where both percentages to be compared were zero. Isolates of *emm* types of unknown *emm* cluster were omitted because the numbers were too small to be meaningful.
(DOCX)

S5 Dataset Microsoft Excel file that was the basis for Fig 4. *Emm* types and *emm* clusters are shown in the left two columns.
(XLSX)

S6 Dataset *Emm* type by *emm* type information. “Other” anatomical sites are excluded, although are taken into account in the “Years Isolated” column.
(DOCX)

S1 Fig Relationship between *emm* type, number of isolates, vaccine class, and anatomical sites in which *emm* types were identified. This encompasses *emm* clusters not included in Fig 5 and Fig 6. The *emm* clusters are indicated on each part of the figure. The font size is proportional to the number of isolates from SSTI infections, when the number of isolates is >9. Pale green: vaccine types; dark green: cross opsonisation-positive, brown: cross opsonisation-equivocal; red: cross opsonisation-negative; yellow: cross opsonisation-unknown.
(PPTX)

S2 Fig *Emm* types isolated from blood. This indicates other sites that the *emm* types were isolated from.
(PPTX)
