# Supplementary material for: Concerns for efficacy of a 30-valent M-protein-based Streptococcus pyogenes vaccine in regions with high rates of rheumatic heart disease
Source: PLoS Negl Trop Dis. 2019 Jul 3;13(7):e0007511. doi: 10.1371/journal.pntd.0007511 (PMC6634427; doi:10.1371/journal.pntd.0007511)
Supplement: S1 Checklist — The checklist used is that designed for observational studies. (DOCX) [file pntd.0007511.s001.docx]

STROBE Statement—checklist of items that should be included in reports of observational studies

|  | Item No. | Recommendation | Page  No. | Relevant text from manuscript |
| --- | --- | --- | --- | --- |
| **Title and abstract** | 1 | (*a*) Indicate the study’s design with a commonly used term in the title or the abstract | 1 | Large scale observational study reveals concerns… |
|  |  | (*b*) Provide in the abstract an informative and balanced summary of what was done and what was found | 2 | It was concluded that the 30mer vaccine is unlikely to have high efficacy in a human population with a high RHD burden. A likely critical issue is protection against emm cluster D4. |
| Introduction | | | |  |
| Background/rationale | 2 | Explain the scientific background and rationale for the investigation being reported | 1-5 | To much to include |
| Objectives | 3 | State specific objectives, including any prespecified hypotheses | 5 | The aims of this study were to understand the Northern Territory S. pyogenes population structure with reference to the emm cluster classification scheme, anatomical site of isolation, and current understanding of the immune response elicited by the candidate 30mer vaccine. |
| Methods | | | |  |
| Study design | 4 | Present key elements of study design early in the paper | 5 | Research activities for ~30 years in the Northern Territory have yielded a substantial collection of S. pyogenes isolates of known emm type, site of isolation, and epidemiological and clinical data.  This study utilised data associated with a large collection of stored GAS isolates, derived from hospital and community-based settings in the Northern Territory. Emm typing data and clinical information were available for 1810 of the isolates in the collection. |
| Setting | 5 | Describe the setting, locations, and relevant dates, including periods of recruitment, exposure, follow-up, and data collection | 6 | Data extracted for each isolate was emm type, date of isolation (with the exception of oneisolate for which the date was not available), anatomical site of isolation, and disease description. Isolates derived from the throat that had an associated disease description of “pharyngitis” were classified as “pharyngitis” isolates. |
| Participants | 6 | (*a*) *Cohort study*—Give the eligibility criteria, and the sources and methods of selection of participants. Describe methods of follow-up  *Case-control study*—Give the eligibility criteria, and the sources and methods of case ascertainment and control selection. Give the rationale for the choice of cases and controls  *Cross-sectional study*—Give the eligibility criteria, and the sources and methods of selection of participants | 5 | This study utilised data associated with a large collection of stored GAS isolates, derived from hospital and community-based settings in the Northern Territory. Emm typing data and clinical information were available for 1810 of the isolates in the collection. These |
|  |  | (*b*) *Cohort study*—For matched studies, give matching criteria and number of exposed and unexposed  *Case-control study*—For matched studies, give matching criteria and the number of controls per case | NA |  |
| Variables | 7 | Clearly define all outcomes, exposures, predictors, potential confounders, and effect modifiers. Give diagnostic criteria, if applicable | 6 | See essentially the whole page |
| Data sources/ measurement | 8* | For each variable of interest, give sources of data and details of methods of assessment (measurement). Describe comparability of assessment methods if there is more than one group |  | This is not really applicable – or the answer is the same as for the previous question |
| Bias | 9 | Describe any efforts to address potential sources of bias | 7 | For some analyses, emm55 isolates were excluded because of their derivation from a large and intensively sampled outbreak (Marshall et al., 2011), with consequent potential to bias the analysis. |
| Study size | 10 | Explain how the study size was arrived at | 5 | This was an observational study, as large as possible. |

Continued on next page

| Quantitative variables | 11 | Explain how quantitative variables were handled in the analyses. If applicable, describe which groupings were chosen and why | N/A | This is not really applicable – the primary data were exclusively in the form of categorical variables, or were dates. All quantitative information was in the form of proportions derived from categorical variables. |
| --- | --- | --- | --- | --- |
| Statistical methods | 12 | (*a*) Describe all statistical methods, including those used to control for confounding | 7 | The significance of differences in proportions were assessed using the Chi squared N-1 test, |
|  |  | (*b*) Describe any methods used to examine subgroups and interactions | throughout | The entire paper is based on the analysis of the sizes of subgroups, as defined by categorical variables. |
|  |  | (*c*) Explain how missing data were addressed | 6 | Date of isolation was unaviailbelf r one out of 1810 isolates. This was simply stated: “with the exception of one isolate for which the date was not available” – and the impact on analysis and conclusions was regarded as insignificant. |
|  |  | (*d*) *Cohort study*—If applicable, explain how loss to follow-up was addressed  *Case-control study*—If applicable, explain how matching of cases and controls was addressed  *Cross-sectional study*—If applicable, describe analytical methods taking account of sampling strategy | 5 | This study utilised data associated with a large collection of stored GAS isolates, derived from hospital and community-based settings in the Northern Territory. Emm typing data and clinical information were available for 1810 of the isolates in the collection. |
|  |  | (*e*) Describe any sensitivity analyses |  |  |
| Results | | | | |
| Participants | 13* | (a) Report numbers of individuals at each stage of study—eg numbers potentially eligible, examined for eligibility, confirmed eligible, included in the study, completing follow-up, and analysed | N/A | This was an analysis of collated data from a large collection of bacterial isolates, the great majority of which had been previously described in publications. |
|  |  | (b) Give reasons for non-participation at each stage | N/A |  |
|  |  | (c) Consider use of a flow diagram | N/A |  |
| Descriptive data | 14* | (a) Give characteristics of study participants (eg demographic, clinical, social) and information on exposures and potential confounders | N/A | This was avoided to the greatest extent possible, primarily for ethical/privacy reasons. We included the minimum data to allow the drawing of the conclusions that we did. |
|  |  | (b) Indicate number of participants with missing data for each variable of interest | 5 | 1: “with the exception of one isolate for which the date was not available” – |
|  |  | (c) *Cohort study*—Summarise follow-up time (eg, average and total amount) | N/A |  |
| Outcome data | 15* | *Cohort study*—Report numbers of outcome events or summary measures over time |  |  |
|  |  | *Case-control study—*Report numbers in each exposure category, or summary measures of exposure |  |  |
|  |  | *Cross-sectional study—*Report numbers of outcome events or summary measures | Throughout, or N/A | The study is based on proportions of categorical variables. |
| Main results | 16 | (*a*) Give unadjusted estimates and, if applicable, confounder-adjusted estimates and their precision (eg, 95% confidence interval). Make clear which confounders were adjusted for and why they were included | N/A | Because we were assessing the significances of differences in proportions defined by categorical variables, P values were used. |
|  |  | (*b*) Report category boundaries when continuous variables were categorized | N/A | We were using proportions defined by categorical variables. |
|  |  | (*c*) If relevant, consider translating estimates of relative risk into absolute risk for a meaningful time period | N/A |  |

Continued on next page

| Other analyses | 17 | Report other analyses done—eg analyses of subgroups and interactions, and sensitivity analyses | N/A |  |
| --- | --- | --- | --- | --- |
| Discussion | | | | |
| Key results | 18 | Summarise key results with reference to study objectives | 17 | The principal aim was to inform prediction of the M protein C-terminal based 30mer vaccine (Dale et al., 2011; Dale et al., 2013) efficacy. It was determined that less than one third of the isolates are cognate with the 30mer vaccine, and for SSTI isolates, the equivalent figure is 25.2%. In the absence of cross protection between emm types, 30mer vaccine efficacy would be likely to be poor. |
| Limitations | 19 | Discuss limitations of the study, taking into account sources of potential bias or imprecision. Discuss both direction and magnitude of any potential bias | 21-22 | The major limitation of this study is that the isolates were not collected systematically but were a convenience sample. However, the high diversity at multiple time points, in combination with evidence for extensive strain persistence, suggests that the isolates do provide a useful picture of S. pyogenes diversity in the Northern Territory over a period of decades. Another limitation is the small number of pharyngitis isolates, although this is in large part a consequence of the low prevalence of pharyngitis in the study area. |
| Interpretation | 20 | Give a cautious overall interpretation of results considering objectives, limitations, multiplicity of analyses, results from similar studies, and other relevant evidence | throughout | We only reported point estimates, and P values on differences on proportions. There was overwhelming evidence for our conclusions, and they were largely in line with current models in this field. |
| Generalisability | 21 | Discuss the generalisability (external validity) of the study results | 21 | We really just note that our findings are similar to others in other study areas – it is just that our study was exceptionally large and over a long period of time. |
| Other information | |  | | |
| Funding | 22 | Give the source of funding and the role of the funders for the present study and, if applicable, for the original study on which the present article is based |  |  |

*Give information separately for cases and controls in case-control studies and, if applicable, for exposed and unexposed groups in cohort and cross-sectional studies.

**Note:** An Explanation and Elaboration article discusses each checklist item and gives methodological background and published examples of transparent reporting. The STROBE checklist is best used in conjunction with this article (freely available on the Web sites of PLoS Medicine at http://www.plosmedicine.org/, Annals of Internal Medicine at http://www.annals.org/, and Epidemiology at http://www.epidem.com/). Information on the STROBE Initiative is available at www.strobe-statement.org.
