# Supplementary material for: Concerns for efficacy of a 30-valent M-protein-based Streptococcus pyogenes vaccine in regions with high rates of rheumatic heart disease
Source: PLoS Negl Trop Dis. 2019 Jul 3;13(7):e0007511. doi: 10.1371/journal.pntd.0007511 (PMC6634427; doi:10.1371/journal.pntd.0007511)
Supplement: S2 Dataset — Part A: Distribution of isolates on the basis of anatomical site of origin. These values were used to construct Fig 1. In Fig 1, the numbers from SSTI and normal skin were combined. 95% CI intervals were calculated, without correction for multiple testing (see Methods). No 95% CI intervals were calculated for the proportions in the bottom row, as these are in large part a reflection of sampling activity, rather than any biological properties of the isolates. Part B: Results of N-1 Chi-suared tests. This experiment addressed differences between isolates from different anatomical sites regarding their distribution into emm clusters. For example, the top left data square is derived from an N-1 Chi-squared test on the percentage of SSTI isolates that are emm cluster A-C1-5 (3% of 1210 isolates), compared with the percentage of blood isolates that are emm cluster A-C1-5 9.9% of 162 isolates). Because of potential for bias, the analysis was also performed with emm55 isolates omitted (starred data points) (see text). We took a conservative approach to assessing the credibility of the differences in proportions. We used the 70 cells in the table to apply a Bonferroni correction. The usual cut-off for significance is P = 0.05. 0.05/70 = 0.00071. Cells coloured red have values <0.0001, which equates to a Bonferroni corrected p value of <0.007, which we regard as strongly supporting the significance of the difference in proportion. Cells coloured orange have p values from 0.0001–0.0007, which equates to Bonferroni corrected p values of 0.007–0.05. We regard this as significant, but less strongly supporting the difference in proportion. The orientations of significant differences are shown using single letters reflecting the first letters of the anatomical sites. A “-”symbol designates where the comparison is of two 0% values. (DOCX) [file pntd.0007511.s003.docx]

**S2 data set. A. Distribution of isolates on the basis of anatomical site of origin.** These values were used to construct Fig.1. In Fig 1, the numbers from SSTI and Normal skin were combined. 95% CI intervals were calculated, without correction for multiple testing (see Methods). No 95% CI intervals were calculated for the proportions in the bottom row, as these are in large part a reflection of sampling activity, rather than any biological properties of the isolates.

|  |  | Total isolates = 1769, No emm55= 1688 | | | | |
| --- | --- | --- | --- | --- | --- | --- |
|  |  | Site of isolation | | | | |
| *Emm* cluster | Cluster total (% all isolates (*95% CI*)) | SSTI (% (*95% CI*)) | Blood (% (*95% CI*)) | Pharyngitis (% (*95% CI*)) | Throat carriage (%( *95% CI*)) | Normal skin (%( *95% CI*)) |
| A-C1-5 | 81 (4.6 (*3.7-5.7*)) | 36 (3.0 (*2.1-4.1*)) | 16 (9.9 (*5.9-15.8*)) | 4 (11.1 (*3.6-27.0))* | 24 (7.3 (*4.8-10.8*)) | 1 (3.1 (*0.2-18.0*)) |
| Clade Y/X | 135 (7.6 (*6.5-9.0*)) | 96 (7.9 (*6.5-9.6*)) | 13 (8.0 (*4.5-13.6*)) | 7 (*19.4 (8.8-36.6*)) | 13 (4.0 (*2.2-6.8*)) | 6 (18.8 (*7.9-37.0*)) |
| D1-5 | 590 (33.4) (*32.2-35.6*)) | 457 (37.8 (*35.0-40.6*)) | 48 (29.6 (*22.9-37.4*)) | 2 (5.7 (*1.0-20.0*)) | 74 (22.5 (*18.2-27.5*)) | 9 (28.1 (*14.4-47.0*)) |
| E1-6 | 840 (47.5 (*45.1-49.8*)) | 547 (45.2 (*42.4-48.0*)) | 83 (51.2 (*43.3-59.1*)) | 23 (63.9 (*46.2-78.7*)) | 171 (52.0 (*46.4-57.5*)) | 16 (50 (*32.2-67.7*)) |
| Outlier | 104 (5.9 (*4.9-7.1*)) | 62 (5.1 (*4.0-6.6*))  21 (1.8 (*1.2-2.8*))* | 2 (1.2 (*0.2-4.9*))  1 (0.6 (*0.03-3.9*))* | 0 | 40 (12.2 (*8.9-16.3*))  1 (0.3 (*0.2-2.2*))* | 0 |
| Unknown | 19 (1.1 (*0.7-1.7*)) | 12 (1.0 (*0.5-1.8*)) | 0 | 0 | 7 (2.1 (*0.9-4.5*)) | 0 |
| Site totals (% all isolates) | **1769** | **1210 (68.4)**  **1169 (69.3)*** | **162 (9.2)**  **161 (9.5)*** | **36**  **(2.0)** | **329 (18.6)**  **290 (17.2)** | **32**  **(1.8)** |

**B.** **Results of** N-1 **Chi-squared** **tests**. This experiment addressed differences between isolates from different anatomical sites regarding their distribution into *emm* clusters. For example, the top left data square is derived from an N-1 Chi-squared test on the percentage of SSTI isolates that are *emm* cluster A-C1-5 (3% of 1210 isolates), compared with the percentage of blood isolates that are *emm* cluster A-C1-5 9.9% of 162 isolates). Because of potential for bias, the analysis was also performed with emm*55* isolates omitted (starred data points) (see text). We took a conservative approach to assessing the credibility of the differences in proportions. We used the 70 cells in the table to apply a Bonferroni correction. The usual cut-off for significance is P = 0.05. 0.05/70 = 0.00071. Cells coloured red have values <0.0001, which equates to a Bonferroni corrected P -value of <0.007, which we regard as strongly supporting the significance of the difference in proportion. Cells coloured orange have P values from 0.0001-0.0007, which equates to Bonferroni corrected P values of 0.007-0.05. We regard this as significant, but less strongly supporting the difference in proportion. The orientations of significant differences are shown suing single letters reflecting the first letters of the anatomical sites. A “-“ symbol designates where the comparison is of two 0% values.

| ***Emm* cluster** | **SSTI vs blood** | **SSTI vs Pharyngitis** | **SSTI vs throat carriage** | **SSTI vs normal skin** | **Blood vs pharyngitis** | **Blood vs throat carriage** | **Blood vs normal skin** | **Pharyngitis vs throat carriage** | **Pharyngitis vs normal skin** | **Throat carriage vs normal skin** |
| --- | --- | --- | --- | --- | --- | --- | --- | --- | --- | --- |
| A-C1-5 | **<0.0001 S<b** | 0.0068 | **0.0004**  **s<tc** | 0.97 | 0.829 | 0.323 | 0.215 | 0.417 | 0.21 | 0.372 |
| Clade Y/X | 0.965 | 0.013 | 0.014 | 0.026 | 0.040 | 0.064 | 0.061 | **0.0001**  **p>tc** | 0.950 | **0.0004**  **tc<ns** |
| D1-5 | 0.042 | **0.0001**  **S<p** | **<0.0001**  **S<tc** | 0.269 | 0.003 | 0.087 | 0.856 | 0.019 | 0.013 | 0.473 |
| E1-6 | 0.150 | 0.027 | 0.028 | 0.591 | 0.168 | 0.868 | 0.902 | 0.175 | 0.251 | 0.829 |
| Outlier | 0.027 | 0.165 | **0.0003**  **S<tc** | 0.190 | 0.510 | **<0.0001**  **b<tc** | 0.535 | 0.027 | **-** | 0.036 |
| Outlier* | 0.262* | 0.417* | 0.060* | 0.444* | 0.642* | 0.632* | 0.661* | 0.724* | **-** | 0.757* |
| Unknown | 0.201 | 0.201 | 0.109 | 0.201 | - | 0.064 | - | 0.381 | - | 0.409 |
