# Supplementary material for: Concerns for efficacy of a 30-valent M-protein-based Streptococcus pyogenes vaccine in regions with high rates of rheumatic heart disease
Source: PLoS Negl Trop Dis. 2019 Jul 3;13(7):e0007511. doi: 10.1371/journal.pntd.0007511 (PMC6634427; doi:10.1371/journal.pntd.0007511)
Supplement: S3 Dataset — Part A. Distribution of the 1810 isolates included in this study into 30mer vaccine protection classes. These data were used to assemble Fig 2. Starred figures are with emm55 isolates omitted. 95% CI’s (see Methods) are provided for the distributions of isolates from different anatomical sites across 30mer protection classes. 95% CI’s are not provided for the distribution of isolates of different 30mer protection classes across sites of isolation, as this is impacted greatly by specimen collection activity, compromising the value of statistical analysis. Part B. This experiment addressed differences between isolates from different anatomical sites regarding their distribution into 30mer vaccine protection classes. For example, the top left data square is derived from an N-1 Chi-squared test on the percentage of SSTI isolates that are 30mer vaccine emm types (25.2% of 1210), vs the percentage of blood isolates that are 30mer vaccine emm types (34.6% of 162 isolates). Starred numbers were calculated with emm55 isolates omitted. As with S2 Data Set, a Bonferroni correction was applied. In this instance, 76 tests for significance were performed. Cells coloured red have values <0.0001, which equates to a Bonferroni corrected P -value of <0.0076, which we regard as strongly supporting significance. Cells coloured orange have P values from 0.0001–0.0006, which equates to Bonferroni corrected P values of 0.008–0.05. We regard this as significant, but less strongly supporting the difference in proportion. Starred values are with emm55 isolates omitted. The orientations of significant differences are indicated in the data cells, with the single letters representing the first letters of site of isolation. (DOCX) [file pntd.0007511.s004.docx]

**S3 Data Set. A.** Distribution of the 1810 isolates included in this study into 30mer vaccine protection classes. These data were used to assemble Fig 2. Starred figures are with *emm*55 isolates omitted. 95% CI’s (see Methods) are provided for the distributions of isolates from different anatomical sites across 30mer protection classes. 95% CI’s are not provided for the distribution of isolates of different 30mer protection classes across sites of isolation, as this is impacted greatly by specimen collection activity, compromising the value of statistical analysis.

|  | **Emm types→** | **Vaccine** | **Cross opsonisation positive** | **Cross opsonisation equivocal** | **Cross opsonisation negative** | **Cross opsonisation unknown** | ***Total (percent all)*** |
| --- | --- | --- | --- | --- | --- | --- | --- |
|  | ***Coverage class total (percent all)*** | **540 (29.8)** | **305 (16.9)** | **175 (9.7)** | **197 (10.9)**  116 (6.7)* | **593 (32.8)** | **1810**  **1729*** |
| Absolute isolate number | SSTI | 305 | 227 | 122 | 141  100* | 415 | **1210**  **1110*** |
|  | Blood | 56 | 19 | 8 | 5  4* | 74 | **162**  **161*** |
|  | Pharyngitis | 24 | 4 | 2 | 0 | 6 | **36** |
|  | Throat carriage | 132 | 47 | 36 | 44  5* | 70 | **329**  **290*** |
|  | Normal skin | 3 | 3 | 4 | 6 | 16 | **32** |
|  | Other | 20 | 5 | 3 | 1 | 12 | **41** |
|  |  |  |  |  |  |  |  |
| % all isolates from site of isolation (horizontal totals) | SSTI | 25.2 (22.8-27.8) | 18.8 (16.6-21.1) | 10.1 (8.5-12.0) | 11.7 (9.9-13.6)  9.0 (7.4-10.9)* | 34.3 (31.7-37.1) |  |
|  | Blood | 34.6 (27.4-42.5) | 11.7 (7.4-18.0) | 4.9 (2.3-9.8) | 3.1 (1.1-7.4)  2.5* (0.8-6.6) | 45.7 (37.9-53.7) |  |
|  | Pharyngitis | 66.7 (49.0-80.9) | 11.1 (3.6-27.0) | 5.6 (1.0-20.0) | 0 (0-12.0) | 16.7 (7.0-33.5) |  |
|  | Throat carriage | 40.1 (34.8-45.7) | 14.3 (10.8-18.7) | 10.9 (7.9-14.9) | 13.4 (10.0-17.6)  1.7* (0.6-4.2) | 21.3 (17.1-26.2) |  |
|  | Normal skin | 9.4 (2.5-26.2) | 9.4 (2.5-26.2) | 12.5 (4.1-29.9) | 18.8 (7.9-37.0) | 50.0 (32.2-67.7) |  |
|  | Other | 48.9 (33.2-64.6) | 12.2 (4.6-27.0) | 7.3 (1.9-21.1) | 2.4 (0.1-14.4) | 29.3 (16.7-45.7) |  |
|  |  |  |  |  |  |  |  |
| % all isolates of coverage class (vertical totals) | SSTI | 56.5 | 74.4 | 69.7 | 71.6  86.2* | 70.0 |  |
|  | Blood | 10.4 | 6.2 | 4.6 | 2.5  3.4 | 12.5 |  |
|  | Pharyngitis | 4.4 | 1.3 | 1.1 | 0 | 1.0 |  |
|  | Throat carriage | 24.4 | 15.4 | 20.6 | 22.3  4.3* | 11.8 |  |
|  | Normal skin | 0.6 | 1.0 | 2.3 | 3.0 | 2.7 |  |
|  | Other | 3.7 | 1.6 | 1.7 | 0.5 | 2.0 |  |

**B. Results of** N-1 **Chi-squared** **tests**. This experiment addressed differences between isolates from different anatomical sites regarding their distribution into 30mer vaccine protection classes. For example, the top left data square is derived from an N-1 Chi-squared test on the percentage of SSTI isolates that are 30mer vaccine *emm* types (25.2% of 1210), vs the percentage of blood isolates that are 30mer vaccine emm types (34.6% of 162 isolates). Starred numbers were calculated with *emm*55 isolates omitted. As with S2 Data Set, a Bonferroni correction was applied. In this instance, 76 tests for significance were performed. Cells coloured red have values <0.0001, which equates to a Bonferroni corrected P -value of <0.0076, which we regard as strongly supporting significance. Cells coloured orange have P values from 0.0001-0.0006, which equates to Bonferroni corrected P values of 0.008-0.05. We regard this as significant, but less strongly supporting the difference in proportion. Starred values are with *emm*55 isolates omitted. The orientations of significant differences are indicated in the data cells, with the single letters representing the first letters of site of isolation.

|  | **Vaccine** | **Cross opsonisation positive** | **Cross opsonisation equivocal** | **Cross opsonisation negative** | **Cross opsonisation unknown** |
| --- | --- | --- | --- | --- | --- |
| SSTI vs blood | 0.011 | 0.027 | 0.034 | 0.0009 | 0.005 |
|  |  |  |  | 0.0049* |  |
| SSTI vs pharyngitis | **<0.0001 S<p** | 0.242 | 0.375 | 0.029 | 0.028 |
|  |  |  |  | 0.060* |  |
| SSTI vs throat carriage | **<0.0001 S<tc** | 0.059 | 0.672 | 0.401 | **<0.0001 S>tc** |
|  |  |  |  | **<0.0001***  **S>tc** |  |
| SSTI vs normal skin | 0.041 | 0.177 | 0.658 | 0.221 | 0.066 |
|  |  |  |  | 0.060* |  |
| SSTI vs other | 0.0007 | 0.286 | 0.557 | 0.060 | 0.507 |
|  |  |  |  | 0.142* |  |
| blood vs pharyngitis | **0.0004 b<p** | 0.919 | 0.862 | 0.286 | 0.0014 |
|  |  |  |  | 0.339* |  |
| blood vs throat carriage | 0.239 | 0.629 | 0.029 | **0.0004 b<tc** | **<0.0001 b>tc** |
|  |  |  |  | 0.560* |  |
| blood vs normal skin | 0.005 | 0.708 | 0.103 | **0.0005 b<ns** | 0.657 |
|  |  |  |  | **0.0002* b<ns** |  |
| blood vs other | 0.092 | 0.929 | 0.544 | 0.813 | 0.058 |
|  |  |  |  | 0.971* |  |
| pharyngitis vs throat carriage | 0.002 | 0.600 | 0.323 | 0.019 | 0.520 |
|  |  |  |  | 0.431* |  |
| pharyngitis vs normal skin | **<0.0001 p>ns** | 0.819 | 0.321 | 0.0068 | 0.0036 |
| pharyngitis vs other | 0.118 | 0.882 | 0.764 | 0.353 | 0.200 |
|  |  |  |  | 0.150 |  |
| throat carriage vs normal skin | **0.0006 tc>ns** | 0.443 | 0.783 | 0.400 | **0.0003 tc<ns** |
|  |  |  |  | **<0.0001* tc<ns** |  |
| throat carriage vs other | 0.281 | 0.715 | 0.479 | 0.043 | 0.246 |
|  |  |  |  | **0.751** |  |
| normal skin vs other | **0.0003 ns<o** | 0.706 | 0.457 | 0.019 | 0.073 |
