# Supplementary material for: Concerns for efficacy of a 30-valent M-protein-based Streptococcus pyogenes vaccine in regions with high rates of rheumatic heart disease
Source: PLoS Negl Trop Dis. 2019 Jul 3;13(7):e0007511. doi: 10.1371/journal.pntd.0007511 (PMC6634427; doi:10.1371/journal.pntd.0007511)
Supplement: S4 Dataset — Part A. Relationship between 30mer vaccine protection class and emm cluster for SSTI isolates. Starred numbers are with emm55 isolates omitted. 95% CI’s are included for the percent values calculated with reference to the “vertical totals”; the number of isolates in each 30mer vaccine protection class. These are the values used for the analysis in Part B below, and also which were visualised in Fig 3. The percent values with reference to anatomical site (horizontal totals) will be in large part a function of specimen collection activity, so have not been subjected to statistical analysis. Part B. Results of N-1 Chi-squared tests. This experiment addressed differences between SSTI isolates of different emm cluster categories their distribution into 30mer vaccine protection classes. For example, the top left data square is derived from an N-1 Chi-squared test on the percentage of “vaccine emm type” isolates that are also “emm cluster A-C1-5” isolates (7.9% of 305 isolates) vs percentage of “cross-opsonisation positive” isolates that are also “emm cluster A-C1-5” isolates (0% of 227 isolates). Starred numbers were calculated with emm55 isolates omitted. As with S2 Data Set and S3 Data Set, a Bonferroni correction was applied. In this instance, 59 tests for significance were performed. Cells coloured red have p values <0.0001, which equates to a Bonferroni corrected P -value of <0.006, which we regard as strongly supporting significance. Cells coloured orange have P values from 0.0001–0.0008, which equates to Bonferroni corrected p values of ~0.006–0.05. We regard this as significant, but less strongly supporting the difference in proportion. Starred values are with emm55 isolates omitted. The orientations of significant differences are indicated in the data cells, with the single letters either designating “vaccine” (v), or the first letter of the cross-opsonisation descriptor. A “-”symbol represents where both percentages to be compared were zero. Isolates of emm type [file pntd.0007511.s005.docx]

**S4 Data Set. A.** Relationship between 30mer vaccine protection class and *emm* cluster for SSTI isolates. Starred numbers are with *emm55* isolates omitted. 96% CI’s are included for the % values calculated with reference to the “vertical totals”; the number of isolates in each 30mer vaccine protection class. These are the values used for the analysis in Part B below, and also which were visualised in Fig 3. The percent values with reference to anatomical site (horizontal totals) will be in large part a function of specimen collection activity, so have not been subjected to statistical analysis.

| **Pyoderma isolates**  **1210 total isolates**  **1169* total isolates without *emm*55** | *Emm* types→ | **Vaccine** | **Cross opsonisation positive** | **Cross opsonisation equivocal** | **Cross opsonisation negative** | **Cross opsonisation unknown** | ***Row total (percent all) (% all without emm55*)*** |
| --- | --- | --- | --- | --- | --- | --- | --- |
| Absolute isolate number | A-C1-5 | 24 | 0 | 0 | 0 | 12 | **36 (3.0) (3.1)*** |
|  | Clade Y/X | 60 | 22 | 0 | 0 | 14 | **96 (7.9) (8.2)*** |
|  | D1-5 | 0 | 81 | 85 | 82 | 209 | **457 (37.8) (39.1)*** |
|  | E1-6 | 221 | 124 | 34 | 18 | **150** | **547 (45.2) (46.8)*** |
|  | Outlier | 0 | 0 | 3 | 41 | 18 | **62 (5.1)** |
|  | Outlier* |  |  |  | 0* |  | **21 (1.8)*** |
|  | unknown | 0 | 0 | 0 | 0 | **12** | **12 (1.0) (1.0)*** |
| ***Protection class totals (%) (% all without emm55*)*** |  | **305 (25.2) (26.1)*** | **227 (18.8) (19.4)*** | **122 (10.1) (10.4)** | **141 (11.7)** | **415 (34.3) (35.5)** |  |
|  |  |  |  |  | **100 (8.6)*** |  |  |
|  |  |  |  |  |  |  |  |
| % all isolates in protection class (vertical totals) | A-C1-5 | 7.9 (5.4-11.4) | 0 | 0 | 0 | 2.9 (1.7-5-5.0) |  |
|  | Clade Y/X | 19.7 (15.6-24.5) | 9.7 (6.3-14.5) | 0 | 0 | 3.4 (2.0-5.6) |  |
|  | D1-5 | 0 | 35.7 (29.7-42.1) | 69.7 (61.0-77.1) | 58.2 (49.9-66.0) | 50.4 (45.6-55.1) |  |
|  |  |  |  |  | 82.0 (72-8-88.7)* |  |  |
|  | E1-6 | 72.5 (67.0-77.3) | 54.6 (48.1-61.2) | 27.9 (20.7-36.4) | 12.8 (8,2-19.3) | 36.1 (31.7-40.9) |  |
|  |  |  |  |  | 18.0 (11.7-26.7)* |  |  |
|  | Outlier | 0 | 0 | 2.5 (0.3-5.8) | 29.1 (22.2-37.1) | 4.3 (2.8-6.8) |  |
|  | Outlier* |  |  |  | 0* |  |  |
|  | unknown | 0 | 0 | 0 | 0 | 2.9 (1.6-5.1) |  |
|  |  |  |  |  |  |  |  |
|  |  |  |  |  |  |  |  |
| % all isolates in cluster (horizontal totals) | A-C1-5 | 66.7 | 0 | 0 | 0 | 33.3 |  |
|  | Clade Y/X | 62.5 | 22.9 | 0 | 0 | 14.6 |  |
|  | D1-5 | 0 | 17.7 | 18.6 | 17.9 | 45.7 |  |
|  | E1-6 | 40.4 | 22.7 | 6.2 | 3.3 | 27.4 |  |
|  | Outlier | 0 | 0 | 4.8 | 66.2 | 29.0 |  |
|  | Outlier* |  |  | 14.3* | 0* | 85.7 |  |
|  | unknown | 0 | 0 | 0 | 0 | 100 |  |

**B. Results of** N-1 **Chi-squared** **tests**. This experiment addressed differences between SSTI isolates of different *emm* cluster categories their distribution into 30mer vaccine protection classes. For example, the top left data square is derived from an N-1 Chi-squared test on the percentage of “vaccine *emm* type” isolates that are also “*emm* cluster A-C1-5” isolates (7.9% of 305 isolates) vs percentage of “cross-opsonisation positive” isolates that are also “*emm* cluster A-C1-5” isolates (0% of 227 isolates). Starred numbers were calculated with *emm*55 isolates omitted. As with S2 Data Set and S3 Data Set, a Bonferroni correction was applied. In this instance, 59 tests for significance were performed. Cells coloured red have values <0.0001, which equates to a Bonferroni corrected P -value of <0.006, which we regard as strongly supporting significance. Cells coloured orange have P values from 0.0001-0.0008, which equates to Bonferroni corrected P values of ~0.006-0.05. We regard this as significant, but less strongly supporting the difference in proportion. Starred values are with *emm*55 isolates omitted. The orientations of significant differences are indicated in the data cells, with the single letters either designating “vaccine” (v), or the first letter of the cross-opsonisation descriptor. A “-“ symbol represents where both percentages to be compared were zero. Isolates of *emm* types of unknown *emm* cluster were omitted because the numbers were too small to be meaningful.

|  | **Vaccine vs CO pos^1^** | **Vaccine vs CO equ^2^** | **Vaccine vs CO neg^3^** | **Vaccine vs CO unkn^4^** | **CO pos vs CO equ** | **CO pos vs CO neg** | **CO pos vs CO unkn** | **CO equ vs CO neg** | **CO equ vs CO unkn** | **CO neg vs CO unkn** |
| --- | --- | --- | --- | --- | --- | --- | --- | --- | --- | --- |
| A-C1-5 | <0.0001  v>p | 0.0014 | 0.0006  v>n | 0.0002 | - | - | 0.0097 | - | 0.057 | 0.041 |
|  |  |  | 0.0038* |  |  |  |  |  |  | 0.085* |
| Clade Y/X | 0.0016 | **<0.0001**  **v>e** | **<0.0001**  **v>n** | **<0.0001**  **v>u** | 0.0004  c>e | 0.0001  p>n | 0.0009 | - | 0.039 | 0.027 |
|  |  |  | **<0.0001***  **v>n** |  |  | 0.0013* |  |  |  | 0.062* |
| D1-5 | **<0.0001**  **v<p** | **<0.0001**  **v<e** | **<0.0001**  **v<n** | **<0.0001**  **v<u** | **<0.0001**  **p<e** | **<0.0001**  **p<n** | 0.0004  p<u | 0.054 | 0.0002  e>u | 0.109 |
|  |  |  | **<0.0001***  **v<n** |  |  | **<0.0001***  **p<n** |  | 0.035* |  | **<0.0001***  **n>u** |
| E1-6 | **<0.0001**  **v>p** | **<0.0001**  **v>e** | **<0.0001**  **v>n** | **<0.0001**  **v>u** | **<0.0001**  **p>e** | **<0.0001**  **p>n** | **<0.0001**  **p>u** | 0.0022 | 0.094 | **<0.0001**  **n<u** |
|  |  |  | **<0.0001***  **v>n** |  |  | **<0.0001***  **p>n** |  | 0.084* |  | **0.0005***  **n<u** |
| Outlier | - | 0.0056 | **<0.0001**  **v<n** | 0.0002  v<u | 0.017 | **<0.0001**  **p<n** | 0.0015 | **<0.0001**  e<n | 0.367 | **<0.0001**  **n>u** |
| Outlier* |  |  | **-*** |  |  | **-*** |  | 0.112* |  | 0.035* |

^1^Cross opsonisation positive, ^2^ Cross opsonisation equivocal, ^3^ Cross opsonisation negative, ^4^ Cross opsonisation unknown
