# Supplementary material for: Concerns for efficacy of a 30-valent M-protein-based Streptococcus pyogenes vaccine in regions with high rates of rheumatic heart disease
Source: PLoS Negl Trop Dis. 2019 Jul 3;13(7):e0007511. doi: 10.1371/journal.pntd.0007511 (PMC6634427; doi:10.1371/journal.pntd.0007511)
Supplement: S6 Dataset — “Other” anatomical sites are excluded, although are taken into account in the “Years Isolated” column. (DOCX) [file pntd.0007511.s007.docx]

**S6 Dataset.** *Emm* type by *emm* type information. “Other sites” are excluded, although are taken into account in the “Years Isolated” column

**Vaccine *emm* types**

| ***emm* type** | ***emm* cluster** | **Numbers of isolates** | | | | | **Years isolated** |
| --- | --- | --- | --- | --- | --- | --- | --- |
|  |  | **SSTI** | **Blood** | **Throat carriage** | **Pharyngitis** | **Normal skin** |  |
| 14 | Clade_Y_3 | 42 | 3 | 7 | 2 | 0 | 1995, 1996, 1997, 1999, 2004, 2005 |
| 11 | E6 | 36 | 0 | 19 | 2 | 1 | 1989, 1995, 1996, 1999, 2001, 2003, 2004, 2005, 2006, 2007 |
| 44 | E3 | 27 | 6 | 12 | 1 | 0 | 1980, 1995, 1996, 1999, 2001, 2002, 2005, 2006, 2011, 2013, 2015 |
| 78 | E1 | 24 | 0 | 15 | 0 | 0 | 1996, 2004, 2005 |
| 58 | E3 | 19 | 6 | 10 | 0 | 0 | 1991, 1992, 1994, 1995, 1996, 1997, 2001, 2004, 2005, 2011, 2012 |
| 1 | A-C3 | 19 | 1 | 18 | 1 | 1 | 1988, 1991, 1995, 1996, 1999, 2004, 2005 |
| 4 | E1 | 18 | 3 | 3 | 3 | 0 | 1990, 1991, 1994, 1995, 1996, 1997, 1999, 2005, 2001 |
| 114 | E4 | 16 | 4 | 2 | 0 | 0 | 1996, 1997, 2001, 2002, 2003, 2004, 2005, 2006, 2011, 2012 |
| 87 | E3 | 15 | 1 | 3 | 0 | 0 | 2005, 2006, 2011, 2014 |
| 89 | E4 | 15 | 3 | 2 | 0 | 0 | 1994, 1995, 1999, 2000, 2001, 2005, 2012, 2013 |
| 81 | E6 | 14 | 14 | 4 | 0 | 1 | 1991, 1992, 1995, 1996, 2002, 2003, 2004, 2011, 2012 |
| 19 | Clade_Y_6 | 10 | 0 | 1 | 2 | 0 | 1994, 1995, 2003, 2004 |
| 22 | E4 | 10 | 1 | 17 | 3 | 0 | 1994, 1995, 1999, 2003, 2004, |
| 77 | E2 | 8 | 0 | 2 | 2 | 0 | 1991, 1992, 1994, 1995, 1996, 1997, 1999, 2001, 2004, 2005 |
| 18 | Clade_Y_5 | 7 | 0 | 1 | 0 | 0 | 1999, 2000, 2005 |
| 92 | E2 | 7 | 5 | 1 | 2 | 0 | 1993, 1994, 1995, 1996, 1999, 2011, 2012, 2013 |
| 49 | E3 | 5 | 2 | 1 | 1 | 0 | 1993, 1994, 1995, 1996, 1999, 2000, 2007, 2012 |
| 3 | A-C5 | 5 | 0 | 0 | 0 | 0 | 2000 |
| 82 | E3 | 4 | 1 | 0 | 0 | 0 | 1990, 1999, 2002, 2005, 2006, 2007 |
| 75 | E6 | 3 | 2 | 9 | 1 | 0 | 1991, 1992, 1994, 1997, 1999, 2003, 2004 |
| 24 | Clade_Y_8 | 1 | 1 | 0 | 0 | 0 | 1994, 2007 |
| 6 | Clade_Y_2 | 0 | 1 | 0 | 0 | 0 | 1990 |
| 2 | E4 | 0 | 1 | 0 | 0 | 0 | 1994 |
| 73 | E4 | 0 | 1 | 0 | 0 | 0 | 2013 |
| 12 | A-C4 | 0 | 0 | 5 | 3 | 0 | 2003, 2004 |
| 28 | E4 | 0 | 0 | 0 | 1 | 0 | 1995 |

**Cross opsonisation positive *emm* types**

| ***emm* type** | ***emm* cluster** | **Numbers of isolates** | | | | |  |
| --- | --- | --- | --- | --- | --- | --- | --- |
|  |  | **SSTI** | **Blood** | **Throat carriage** | **Pharyngitis** | **Normal skin** | **Years isolated** |
| 33 | D4 | 27 | 2 | 3 | 0 | 0 | 1993, 1999, 2003, 2004, 2005, 2011 |
| 97 | D5 | 21 | 0 | 3 | 0 | 0 | 1987, 1988, 1994, 1995, 1996, 2001, 2004, 2005, 2006 |
| 183 | E3 | 21 | 1 | 12 | 0 | 0 | 1995, 2004, 2005, 2006 |
| 25 | E3 | 20 | 3 | 4 | 0 | 1 | 1991, 1994, 1995, 1996, 1997, 2003, 2004, 2005 |
| 74 | Cluster_Y_15 | 17 | 2 | 1 | 1 (25) | 0 | 1990, 1993, 1994, 1995, 1996, 1999, 2001, 2002, 2017 |
| 123 | D3 | 17 | 1 | 4 | 0 | 0 | 1991, 1992, 1993, 1998, 1999, 2004, 2005, 2007 |
| 65/69 | E6 | 17 | 2 | 2 | 0 | 0 | 1991, 1995, 1996, 1997, 2006, 2007 |
| 15 | E3 | 17 | 0 | 7 | 0 | 0 | 1995, 2005, 2006 |
| 52 | D4 | 16 | 1 | 1 | 0 | 0 | 1995, 1997 |
| 85 | E6 | 16 | 3 | 2 | 0 | 0 | 1990, 1991, 1992, 1994, 1995, 1999, 2000, 2011, 2012 |
| 76 | E2 | 15 | 1 | 4 | 1 (25) | 0 | 1993, 1995, 1996, 1997, 2004, 2005 |
| 60 | E1 | 5 | 1 | 0 | 0 | 0 | 1996, 1997 |
| 105 | Cluster_Y_16 | 4 | 1 | 3 | 1 (25) | 0 | 2001, 2004, 2004, 2011 |
| 8 | E4 | 5 | 1 | 0 | 0 | 1 | 2002, 2004, 2012 |
| 68 | E2 | 3 | 0 | 1 | 0 | 0 | 1992 |
| 102 | E4 | 3 | 0 | 0 | 0 | 0 | 1991, 1995, 2001 |
| 109 | E4 | 1 | 0 | 0 | 1 (25) | 0 | 1994, 1997 |
| 48 | E6 | 1 | 0 | 0 | 0 | 0 | 1996 |
| 122 | Clade_Y_17 | 1 | **0** | **0** | **0** | 1 | 1991 |

**Cross opsonisation equivocal *emm* types**

| ***emm* type** | ***emm* cluster** | **Numbers of isolates** | | | | | **Years isolated** |
| --- | --- | --- | --- | --- | --- | --- | --- |
|  |  | **SSTI** | **Blood** | **Throat carriage** | **Pharyngitis** | **Normal skin** |  |
| 100 | D2 | 41 | 1 | 13 | 1 | 1 | 1990, 1991, 1994, 1996, 1997, 1999, 2004, 2005, 2006, 2014 |
| 53 | D4 | 29 | 5 | 3 | 0 | 0 | 1990, 1991, 1992, 1995, 1997, 1998, 1999, 2000, 2001, 2004, 2005, 2011, 2012, 2015 |
| 42 | E6 | 23 | 0 | 5 | 1 | 0 | 1994, 1995, 1996, 2000, 2001, 2004, 2005 |
| 71 | D2 | 15 | 0 | 13 | 0 | 0 | 1991, 1997, 1999, 2004, 2005 |
| 63 | E6 | 11 | 1 | 2 | 0 | 1 | 1991, 1997, 1998 |
| 95 | Outlier 2 | 3 | 1 | 0 | 0 | 0 | 1993 |

**Cross opsonisation negative *emm* types**

| ***emm* type** | ***emm* cluster** | **Numbers of isolates** | | | | | **Years isolated** |
| --- | --- | --- | --- | --- | --- | --- | --- |
|  |  | **SSTI** | **Blood** | **Throat carriage** | **Pharyngitis** | **Normal skin** |  |
| 55 | Outlier 1 | 41 | 1 | 39 | 0 | 0 | 1991, 1992, 2005 |
| 54 | D1 | 25 | 1 | 2 | 0 | 0 | 1991, 1992, 1993, 1994, 1999, 2000, 2005, 2007, 2014 |
| 116 | D4 | 24 | 0 | 2 | 0 | 0 | 1992, 1999, 2003, 2004, 2005, 2007, 2008 |
| 70 | D4 | 20 | 2 | 0 | 0 | 1 | 1991, 1992, 1995, 1998, 1999, 2000, 2001, 2004, 2005, 2006 |
| 124 | E4 | 18 | 0 | 1 | 0 | 0 | 1991, 1997, 2002, 2005 |
| 80 | D4 | 13 | 1 | 0 | 0 | 0 | 1995, 1996, 1997 |

**Cross opsonisation unknown *emm* types**

| ***emm* type** | ***emm* cluster** | **Numbers of isolates** | | | | | **Years isolated** |
| --- | --- | --- | --- | --- | --- | --- | --- |
|  |  | **SSTI** | **Blood** | **Throat carriage** | **Pharyngitis** | **Normal skin** |  |
| 101 | D4 | 62 | 7 | 5 | 1 | 0 | 1990, 1991, 1994 1996, 1997, 1998, 2004, 2005, 2006, 2007, 2012 |
| 91 | D4 | 47 | 6 | 7 | 0 | 4 | 1991, 1993, 1994, 1995, 1997, 1999, 2001, 2002, 2004, 2005, 2006, 2011, 2012, 2014 |
| 99 | E6 | 35 | 0 | 1 | 1 | 0 | 1990, 1994, 1997, 1998, 1999, 2001, 2004, 2005, 2006, 2007 |
| 232 | E4 | 17 | 3 | 5 | 0 | 0 | 1992, 1993, 1995, 1997, 1999, 2001, 2004, 2005, |
| 56 | D4 | 16 | 4 | 0 | 0 | 0 | 1991, 1993, 1994, 1995, 2004, 2005 |
| 13L | E2 | 16 | 0 | 0 | 0 | 0 | 1997, 1998, 2005, 2006 |
| 9 | E3 | 15 | 1 | 0 | 0 | 0 | 1993, 1994, 1995, 1999, 2001 |
| 225 | D4 | 14 | 0 | 1 | 0 | 0 | 1994, 1995, 2000, 2001 |
| 221 | Outlier 5 | 14 | 0 | 0 | 0 | 0 | 1995, 1996 |
| 90 | E2 | 13 | 2 | 0 | 1 | 0 | 1995, 1996, 1997, 1999 |
| 103 | E3 | 13 | 0 | 14 | 0 | 0 | 1992, 2003, 2004, 2005 |
| 230 | D4 | 12 | 3 | 9 | 0 | 0 | 1997, 2004, 2005, 2011, 2013 |
| 193 | A-C4 | 11 | 1 | 1 | 0 | 0 | 1994, 1995 |
| 57 | Clade Y_14 | 11 | 2 | 0 | 1 | 4 | 1991, 1997 |
| 98 | D4 | 11 | 2 | 2 | 0 | 0 | 1987, 1990, 1991, 1992, 1993, 1996, 1997, 2002, 2003, 2004, 2005, 2011 |
| 192 | D4 | 11 | 4 | 4 | 0 | 0 | 1999, 2000, 2001 |
| 110 | E2 | 11 | 1 | 4 | 1 | 0 | 1992, 1995, 1996, 1997, 1999, 2001, 2003, 2004, 2005 |
| stG653 | unknown | 11 | 0 | 7 | 0 | 0 | 2005 |
| 207 | D1 | 10 | 4 | 1 | 0 | 0 | 1991, 1992, 1994, 1995, 1997, 2011 |
| 108 | D4 | 10 | 3 | 0 | 0 | 1 | 1991, 1996, 2002, 2005, 2012, 2014 |
| 88 | E4 | 10 | 1 | 0 | 0 | 2 | 1991, 2014, 2015 |
| 165 | E1 | 7 | 0 | 2 | 0 | 0 | 1997 |
| 86 | D4 | 6 | 0 | 0 | 0 | 0 | 1997, 1998, 1999, 2002 |
| 67 | E4 | 5 | 0 | 0 | 0 | 2 | 1991, 1992 |
| 104 | E2 | 4 | 1 | 2 | 0 | 0 | 1999, 2000, 2012, 2014 |
| 217 | D3 | 4 | 0 | 0 | 0 | 0 | 1998, 1999 |
| 41 | D4 | 4 | 1 | 1 | 0 | 0 | 1995, 1997, 2007 |
| 222 | Outlier 6 | 4 | 0 | 1 | 0 | 0 | 1996, 1997 |
| 233 | Clade_Y_21 | 2 | 1 | 0 | 0 | 0 | 1992, 1995, 1998 |
| 106 | E2 | 1 | 1 | 0 | 0 | 0 | 1992, 1993, 1994 |
| 178 | D4 | 1 | 0 | 0 | 0 | 0 | 2007 |
| 231 | E3 | 1 | 0 | 0 | 0 | 0 | 1992 |
| 39 | A-C4 | 1 | 0 | 0 | 0 | 0 | 2002, 2012 |
| 112 | E4 | 1 | 0 | 0 | 1 | 0 | 1993, 1994 |
| stGrobn | unknown | 1 | 0 | 0 | 0 | 0 | 2014 |
| 93 | D4 | 1 | 0 | 0 | 0 | 0 | 1993 |
| 144 | E3 | 1 | 0 | 0 | 0 | 0 | 1998 |
| 218 | Clade_Y_20 | 1 | 1 | 1 | 0 | 1 | 1991 |
| 197 | A-C2 | 0 | 12 | 0 | 0 | 0 | 2012 |
| 113 | E3 | 0 | 10 | 0 | 0 | 0 | 2011, 2012, 2013 |
| 142 | A-C1 | 0 | 1 | 0 | 0 | 0 | 2001 |
| 229 | A-C4 | 0 | 1 | 0 | 0 | 0 | 1994 |
| 236 | Clade_X_4 | 0 | 1 | 0 | 0 | 0 | 2013 |
| 166 | E2 | 0 | 0 | 1 | 0 | 0 | 1997 |
| 170 | E5 | 0 | 0 | 1 | 0 | 0 | 1999 |
| 117 | E2 | 0 | 0 | 0 | 0 | 2 | 1991 |
