# Supplementary material for: Concerns for efficacy of a 30-valent M-protein-based Streptococcus pyogenes vaccine in regions with high rates of rheumatic heart disease
Source: PLoS Negl Trop Dis. 2019 Jul 3;13(7):e0007511. doi: 10.1371/journal.pntd.0007511 (PMC6634427; doi:10.1371/journal.pntd.0007511)
Supplement: S1 Fig — This encompasses emm clusters not included in Fig 5 and Fig 6. The emm clusters are indicated on each part of the figure. The font size is proportional to the number of isolates from SSTI infections, when the number of isolates is >9. Pale green: vaccine types; dark green: cross opsonisation-positive, brown: cross opsonisation-equivocal; red: cross opsonisation-negative; yellow: cross opsonisation-unknown. (PPTX) [file pntd.0007511.s008.pptx]

## Slide 1
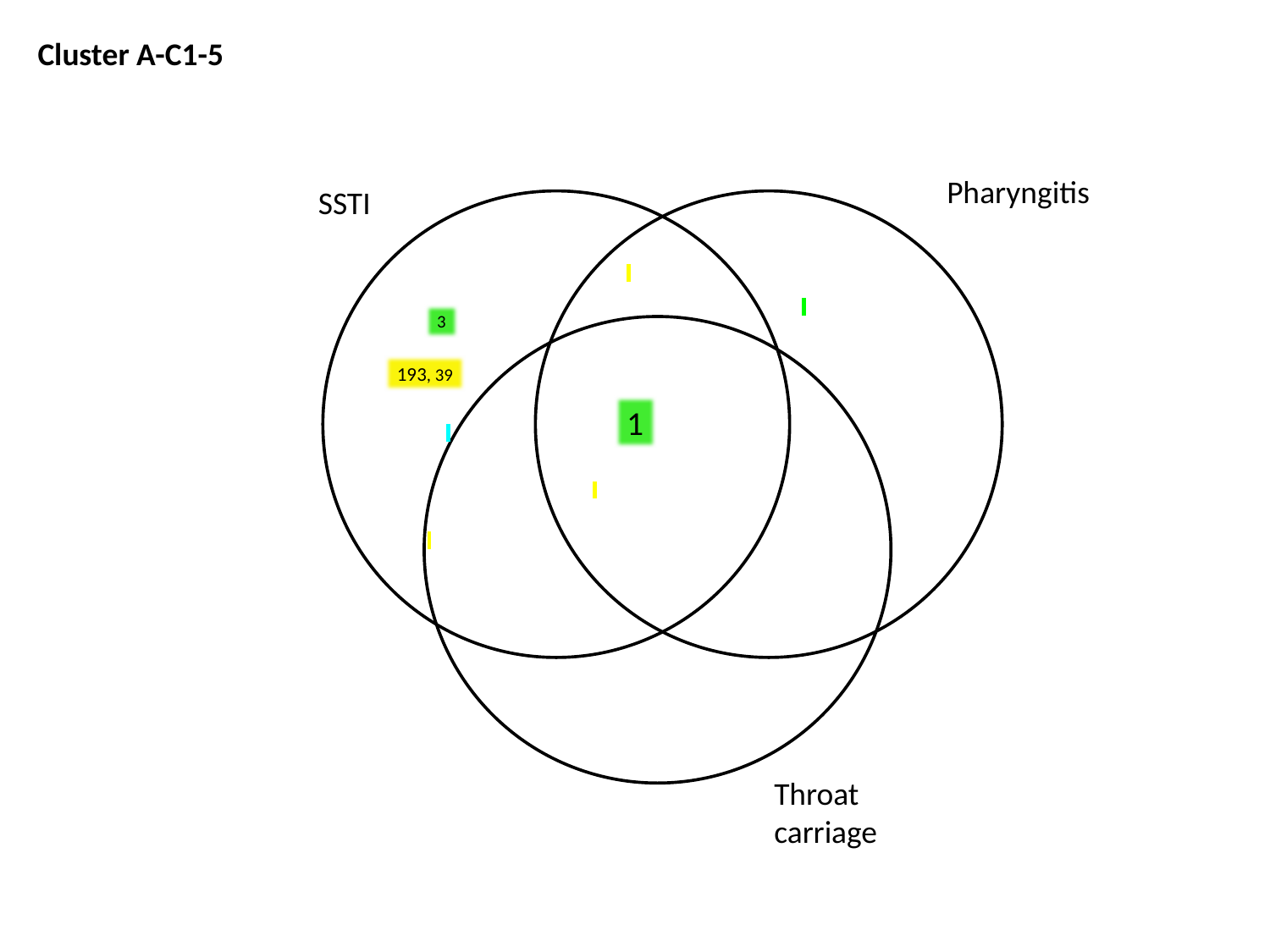

Cluster A-C1-5
Pharyngitis
SSTI
3
193, 39
1
Throat
carriage

## Slide 2
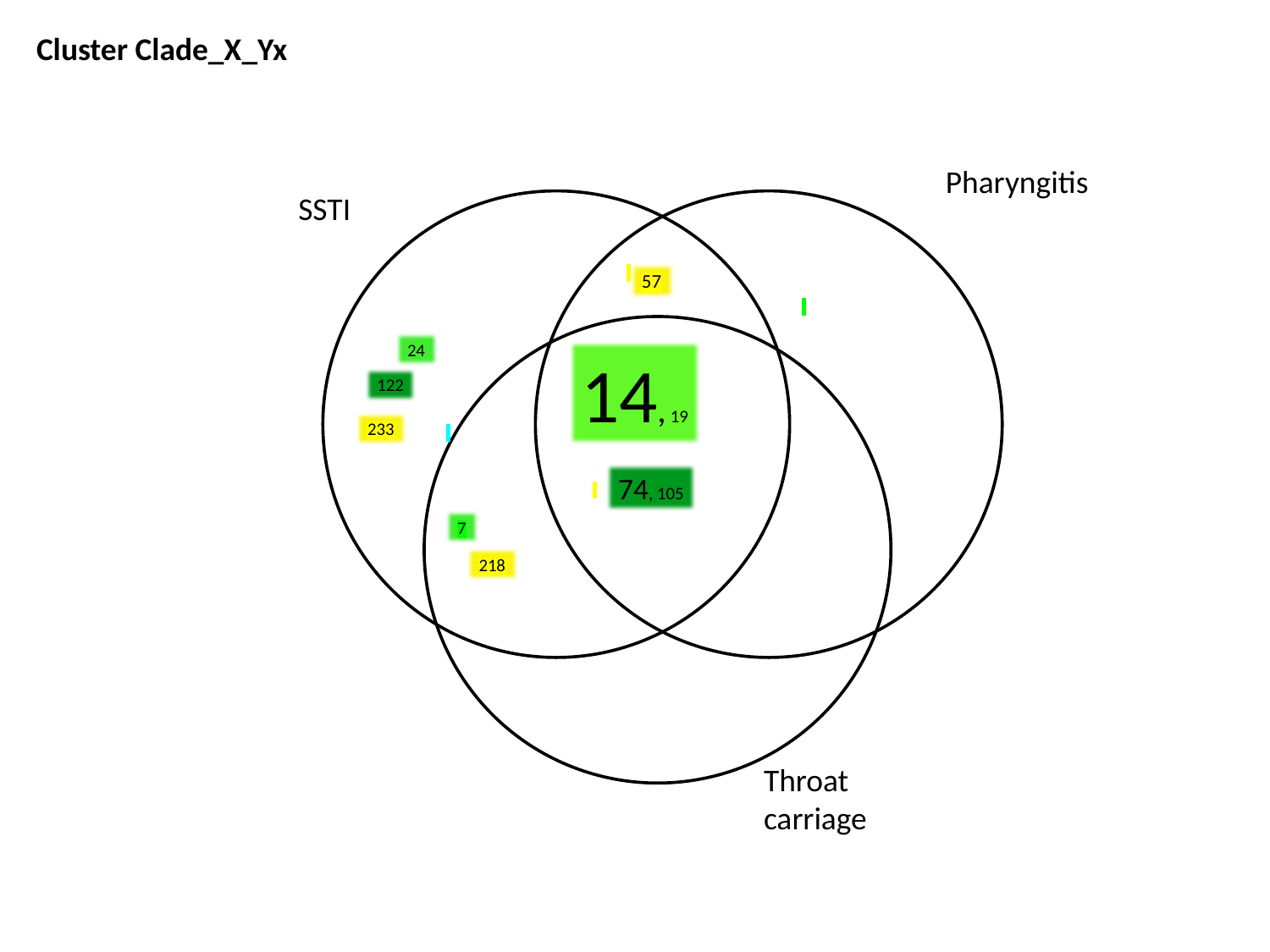

Cluster Clade_X_Yx
Pharyngitis
SSTI
57
24
14, 19
122
233
74, 105
7
218
Throat
carriage

## Slide 3
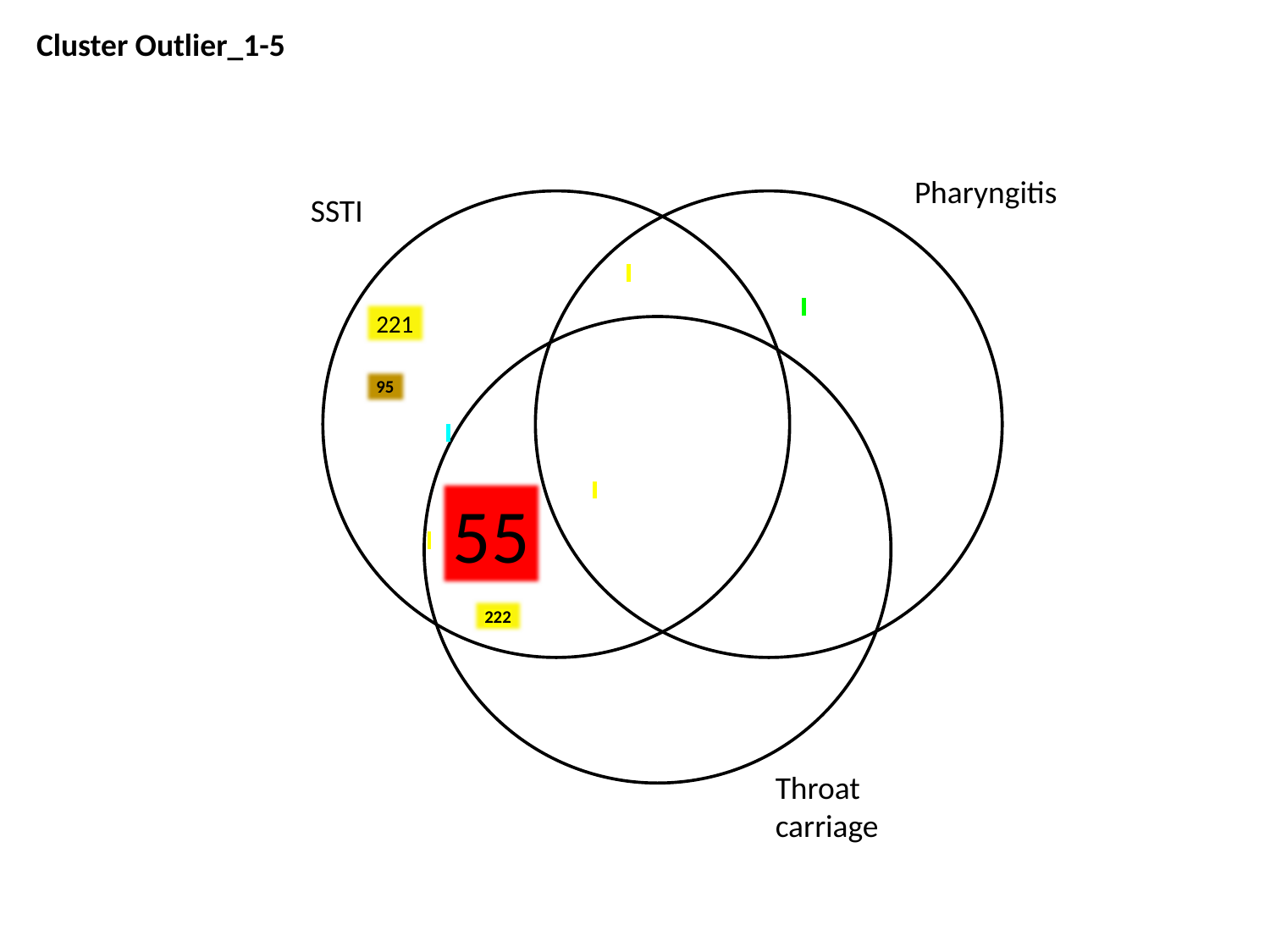

Cluster Outlier_1-5
Pharyngitis
SSTI
221
95
55
222
Throat
carriage

## Slide 4
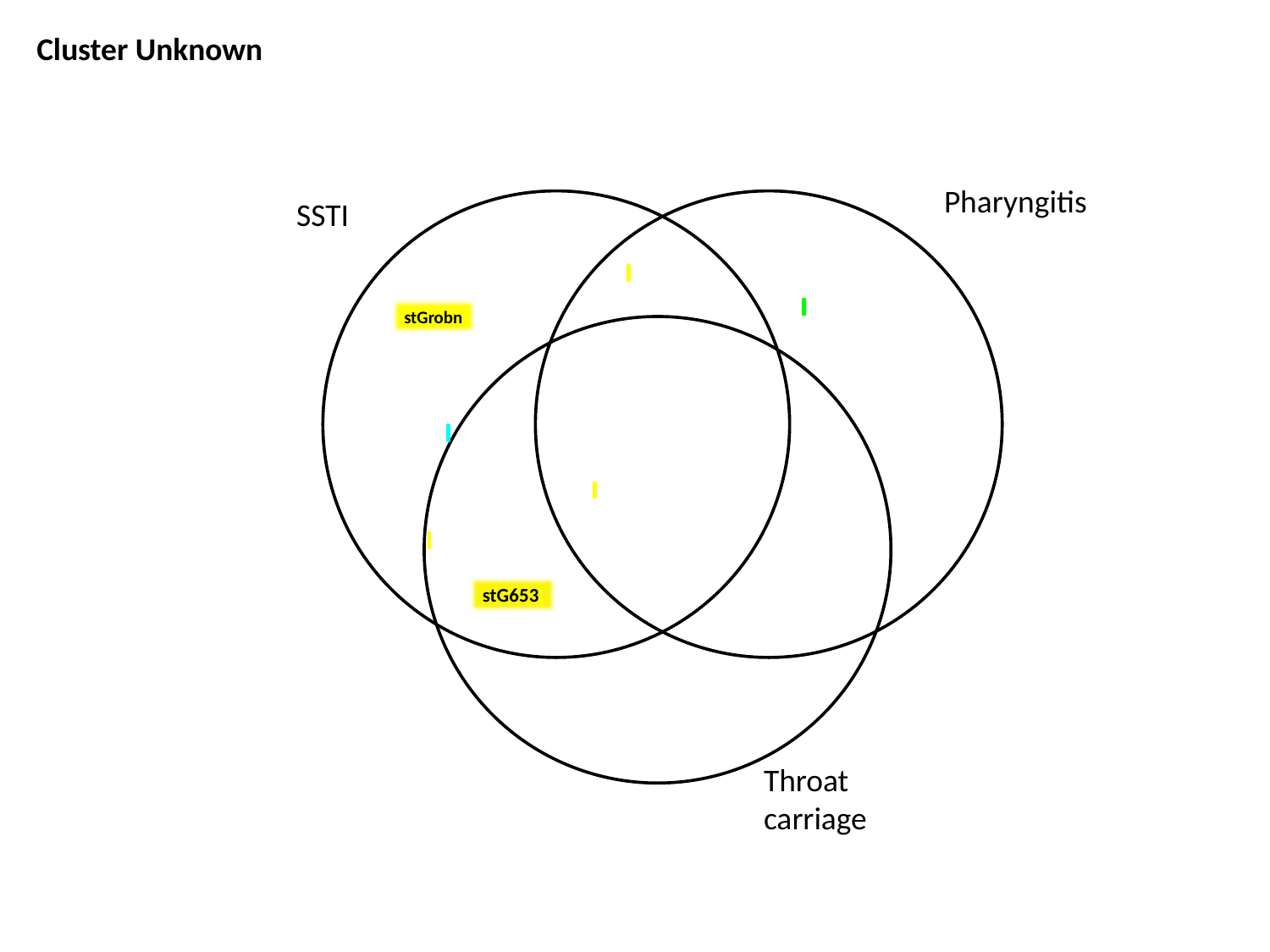

Cluster Unknown
Pharyngitis
SSTI
stGrobn
stG653
Throat
carriage
