# Supplementary figures and images for: Concerns for efficacy of a 30-valent M-protein-based Streptococcus pyogenes vaccine in regions with high rates of rheumatic heart disease
Source: PLoS Negl Trop Dis. 2019 Jul 3;13(7):e0007511. doi: 10.1371/journal.pntd.0007511 (PMC6634427; doi:10.1371/journal.pntd.0007511)

## Slide 1
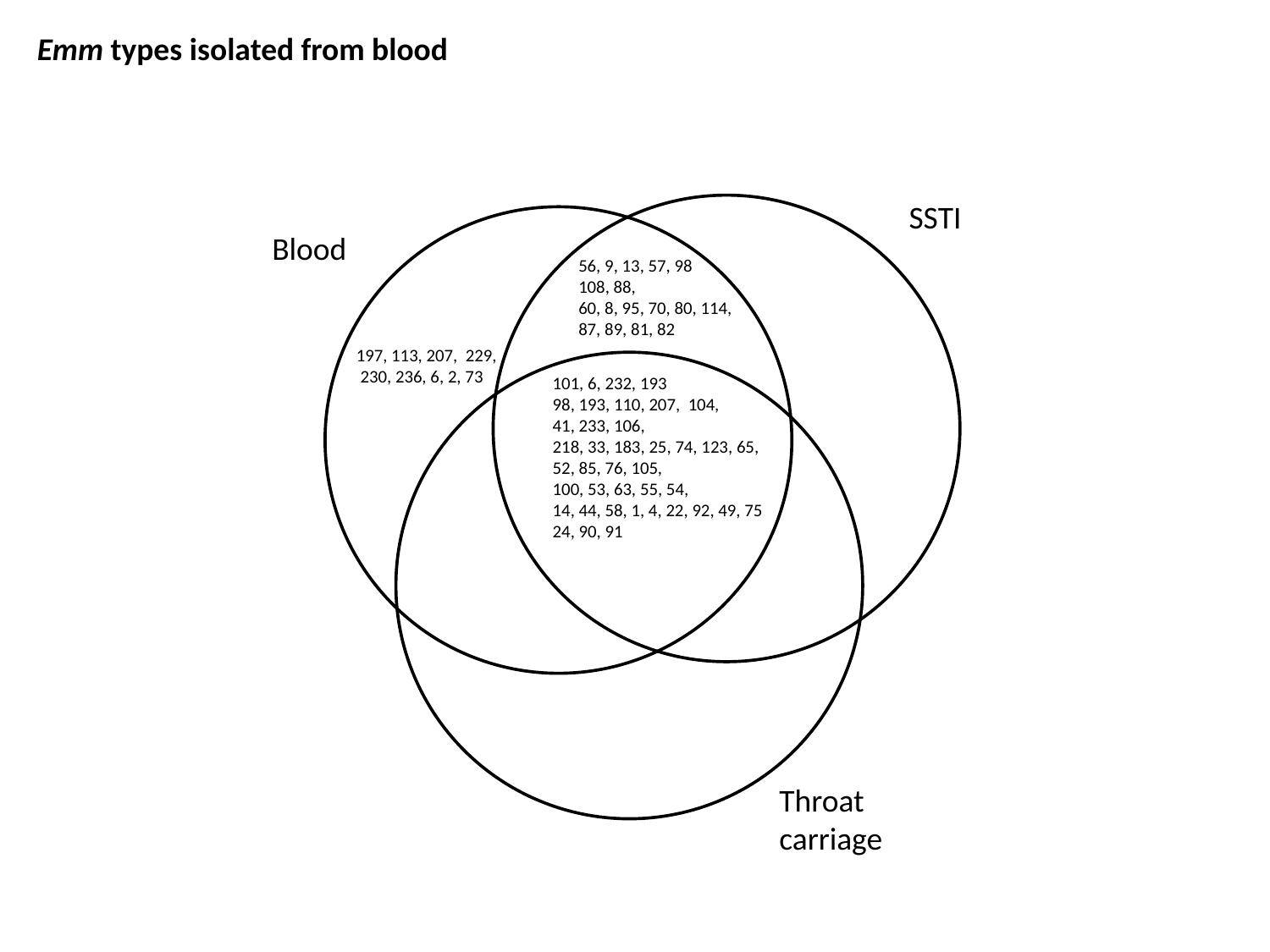

Emm types isolated from blood
SSTI
Blood
56, 9, 13, 57, 98
108, 88,
60, 8, 95, 70, 80, 114,
87, 89, 81, 82
197, 113, 207, 229,
 230, 236, 6, 2, 73
101, 6, 232, 193
98, 193, 110, 207, 104,
41, 233, 106,
218, 33, 183, 25, 74, 123, 65,
52, 85, 76, 105,
100, 53, 63, 55, 54,
14, 44, 58, 1, 4, 22, 92, 49, 75
24, 90, 91
Throat
carriage

Supplement: S2 Fig — This indicates other sites that the emm types were isolated from. (PPTX) [file pntd.0007511.s009.pptx]
